# Novel Syngeneic Cell Lines for Studying High-Risk BRAF^V600E^-Driven Colorectal Cancer *In Vivo*

**DOI:** 10.1158/2767-9764.CRC-25-0599

**Published:** 2026-02-16

**Authors:** Jasmin Traichel, Ariane Metzger, Maria Walz, Reinhild Feuerstein, Nadine Wohlfeil, Patrick Metzger, Lisa Marx, Célia Asdih, Agnes Lindenthal, Alina Seger, Jule Schrimpf, Luigi L. Nardella, Silke Kowar, Ian J. Frew, Sebastian Halbach, Rebecca Kesselring, Melanie Boerries, Ricarda Griffin, Tilman Brummer

**Affiliations:** 1 Institute of Molecular Medicine and Cell Research (IMMZ), Faculty of Medicine, University of Freiburg, Freiburg, Germany.; 2 German Cancer Consortium (DKTK), Partner site Freiburg, a partnership between DKFZ and Medical Center - University of Freiburg, Freiburg, Germany.; 3 Faculty of Biology, University of Freiburg, Freiburg, Germany.; 4 Institute of Medical Bioinformatics and Systems Medicine (IBSM), Faculty of Medicine, Medical Center-University of Freiburg, Freiburg, Germany.; 5 Department of General and Visceral Surgery, Faculty of Medicine, University of Freiburg, Freiburg, Germany.; 6 Centre for Biological Signaling Studies BIOSS, University of Freiburg, Freiburg, Germany.; 7 Department of Internal Medicine I, Hematology, Oncology and Stem Cell Transplantation, Medical Center-University of Freiburg, Faculty of Medicine, University of Freiburg, Freiburg, Germany.

## Abstract

Combining anti-EGFR antibodies with BRAF/MEK/ERK pathway blockade opened new treatment options for BRAF^V600E^-driven colorectal cancer. Nevertheless, responses of these poor prognostic cancers are short-lived and heterogeneous, thereby highlighting the unmet need for novel concepts integrating targeted therapy and immuno-oncology. To this end, an immunocompetent mouse model with reliable disease onset and responding to clinically relevant drugs is essential but unavailable. In this study, we generated three cell lines from peritoneal metastases originating from orthotopically transplanted organoids carrying *Braf*^*V600E*^, *Trp53*^*R172H*^, and *Apc* mutations and characterized their ground state and responses to targeted therapy compounds in detail by RNA sequencing, whole-exome sequencing, and various functional assays. Interestingly, these so-called NaJa lines displayed distinct differentiation states and responses to the clinically relevant RAF inhibitors (RAFi) encorafenib and exarafenib, thereby resembling the clinical heterogeneity of BRAF^V600E^-driven colorectal cancer. RAFi resistance was overcome by the EGFR family inhibitor afatinib. RAFis also influenced the expression of the antigen presentation machinery, cytokines, and other immunomodulatory factors. Upon retransplantation into syngeneic mice, all NaJa lines established aggressive tumors with distinct tumor microenvironments matching their differentiation states. Thus, the NaJa lines provide a unique tool to study tumor heterogeneity, drug resistance, and the interplay between tumor, stroma, and immune cells in BRAF^V600E^-driven colorectal cancer.

**Significance:**

We present three newly isolated syngeneic BRAF^V600E^-driven colorectal cancer cell lines with distinct *in vitro* and *in vivo* phenotypes. They differ in their response to drug treatments, resemble patient tumor heterogeneity, and enable efficient tumor growth in immunocompetent mice for tumor-immune microenvironment studies.

## Introduction

Colorectal cancer poses a significant global socioeconomic problem with an increasing prevalence (1). Colorectal cancer represents a(n) (epi)genetically heterogeneous disease, and its various genomic alterations influence the course of disease, affect prognosis, and steer therapeutic regimen (2). Approximately 10% of colorectal cancers carry mutations in the *BRAF protooncogene*, encoding the serine/threonine protein kinase BRAF, a major signal transducer in the RAS/RAF/MEK/ERK axis, or ERK pathway in short (1). Under physiologic conditions, this pathway becomes activated by a plethora of receptor tyrosine kinases (RTK), such as the EGFR, which in turn activate RAS. RAS recruits RAF kinases to the membrane, resulting in their activation by dimerization followed by phosphorylation of their downstream targets MEK and ERK (3). V600E represents the most common *BRAF* alteration in colorectal cancer and generates a mutation-specific salt bridge, thereby locking the kinase domain in a constitutively active conformation (4). BRAF^V600E^ confers a dismal prognosis due to its aggressive and distinct metastatic spreading pattern, in particular to the peritoneum, and its refractoriness against standard treatments (1, 5). Consequently, a lot of hope was placed on the BRAF^V600E^-selective RAF inhibitors (RAFi) that were developed to target this oncoprotein in melanoma, with the aim to recapitulate their success in colorectal cancer. Unfortunately, however, these type I^1/2^ RAFis, which exploit the specific properties of BRAF^V600E^ and bind to this oncoprotein in its monomeric state, are ineffective in colorectal cancer, both within a monotherapy setting and in combination with MEK inhibitors (MEKi; refs. 3, 6). Insights into the signaling network of BRAF^V600E^-driven colorectal cancers revealed that pharmacologic ERK pathway blockade relieves the EGFR from multiple negative feedback loops, resulting in its reactivation and the activation of multiple signaling pathways contributing to cellular survival and proliferation (7–9). Moreover, the reactivated EGFR fuels RAS-GTP loading, thereby promoting the formation of BRAF dimers in which one protomer is drug-bound and inhibited but, at the same time, allosterically transactivates its binding partner that remains drug-free due to negative allostery. This causes paradoxical ERK pathway activation and blunts the efficacy of type I^1/2^ RAFis (3). Therefore, the type I^1/2^ compounds, vemurafenib, dabrafenib, or encorafenib, were combined in clinical trials, either singly or in combination with MEKis and EGFR-targeting antibodies (1). The most effective combination includes encorafenib, a RAFi with prolonged on-target residency and improved pharmacodynamics (10, 11), with the MEKi binimetinib and the anti-EGFR antibody cetuximab (12). This so-called BEACON protocol represents the approved standard of care for second-line systemic treatment and has even shown promising results in the first line (13). Unfortunately, not all tumors initially respond to this protocol, as objective response rates are below 30% (14–16). This pinpoints to still ill-defined intrinsic resistance mechanisms conferred by private co-mutations or signaling network alterations. For example, ERK pathway inhibition upregulates other RTKs than the EGFR in human colorectal cancer cell lines (7), which are spared by cetuximab, and dynamic RTK signaling network rewiring might contribute to cetuximab resistance in patients (17). Indeed, most patients, who experienced an initial response to the BEACON regimen, usually relapse because of acquired resistance (18). This highlights the need for novel therapeutic concepts, such as the inclusion of immunotherapies, which provide long-term control of residual cancer cells. This approach, however, is confounded by the fact that colorectal cancers, in particular the highly aggressive microsatellite-stable (MSS^+^) BRAF^V600E^-driven subtype, often possess an immunologically “cold” tumor microenvironment (TME; ref. 19). To obtain a deeper mechanistic understanding how these cancers escape immune surveillance and how targeted therapy might interfere with this process, better and fully immunocompetent *in vivo* models will be required to study BRAF^V600E^-driven colorectal cancer and explore novel treatment options for systemic disease. Genetically engineered mouse models (GEMM) provide such an opportunity, and several conditional BRAF^V600E^-expressing *knock-in* models have been established using intestine-specific Cre drivers (20–24). These *in vivo* and *ex vivo* studies, however, revealed that oncogenic BRAF by itself is insufficient to drive malignant transformation, in particular due to its negative impact on the intestinal stem cell (ISC) niche, and requires the cooperation with other (epi)genetic alterations dysregulating the WNT/APC/β-catenin or TGFβ pathways (20, 23, 25–28). Thus, these GEMMs provide a functional explanation for the spectrum of co-mutations observed in BRAF^V600E^-driven colorectal cancers (18, 24, 29). Yet, disease onset and metastatic burden are heterogeneous and unpredictable in these models, thereby complicating the establishment of “preclinical” cohorts to evaluate novel therapeutic strategies combining targeted and immunotherapies. Transplantation of syngeneic cell lines is often used for this purpose and, so far, tumor immunologic aspects of colorectal cancer have been predominantly and successfully studied using two syngeneic cell lines, CT26 and MC38 (30, 31). These models, however, are unsuitable to study BRAF^V600E^-driven colorectal cancer as described in detail in the “Discussion” section. Here, we report the generation of a syngeneic transplantation model for a highly aggressive subset of BRAF^V600E^-driven colorectal cancer that recapitulates many aspects of the human disease, including its heterogeneity, which poses a major but mechanistically ill-defined hurdle in treating metastatic disease.

## Materials and Methods

### Mice and genotyping

The generation of BPAC donors has been described previously (23) and were all maintained on C57BL/6N background (RRID: MGI:2159965). To avoid confusion, it should be mentioned that the murine equivalent of V600 is sometimes referred to as V637, as different but conserved splice variants have been used for the annotation of human and murine BRAF proteins (32). For simplicity and as the human reference transcript does not contain exons of the murine reference transcript, we apply V600E for the V637E mutation throughout the article, as also practiced by others for this and related knock-in alleles (25, 33, 34). C57BL/6N mice (Charles River Laboratory strain) were provided by the local stock of the animal facility of the Freiburg University Medical Center. Mice were kept under specific pathogen-free conditions in the animal facility of the University Medical Center Freiburg according to institutional guidelines. Animals experienced a 12-hour light/dark cycle, received standard diet and water *ad libitum*, and were inspected by animal caretakers on a daily basis.

Genomic DNA was isolated from ear biopsies taken at weaning. The following primers were used for genotyping: *Apc* status: 5′-GTT​CTG​TAT​CAT​GGA​AAG​ATA​GGT​GGT​C-3′, 5′-CAC​TCA​AAA​CGC​TTT​TGA​GGG​TTG​ATT​C-3′, *Braf* status: 5′-TGA​GTA​TTT​TTG​TGG​CAA​CTG​C-3′, 5′-CTC​TGC​TGG​GAA​AGC​GGC-3′, *CreERT2* status: 5′-GCA​TAA​CCA​GTG​AAA​CAG​CAT​TGC​TG-3′, 5′-GGA​CAT​GTT​CAG​GGA​TCG​CCA​GGC​G-3′, 5′-GAG​ACT​CTG​GCT​ACT​CAT​C-3′, 5′-CCT​TCA​GCA​AGA​GCT​GGG​GAC-3′, and *Trp53* status: 5′-AGC​CTT​AGA​CAT​AAC​ACA​CGA​ACT-3′, 5′-CTT​GGA​GAC​ATA​GCC​ACA​CTG-3′, 5′-GCC​ACC​ATG​GCT​TGA​GTA​A-3′.

### Ethics approval

Animal experimentation was carried out according to the German law for animal protection and was approved by the government commission for animal protection and the local ethics committee (G-17/101, G-20/172, and X-19/05C).

### Organoid culture

Colonic organoids were generated from sacrificed mice carrying the *Braf*^*floxV600E*^, *Trp53*^*LSL-R172H*^, and *Apc*^*flox*^ alleles and *Villin*::CreER^T2^ transgene as described previously (23). In brief, the organoids were plated in 30 μL Matrigel domes and incubated at 37°C. The Matrigel domes were overlaid with 500 μL complete culture medium (Advanced DMEM/F12 with 2 mmol/L L-glutamine, 10 mmol/L HEPES, 100 U/mL penicillin, 100 μg/mL streptomycin, 1 × N2 and 1 × B27 supplements (both from Thermo Fisher Scientific), 81.5 mg/mL N-acetylcysteine (Sigma-Aldrich), 100 ng/mL murine recombinant Noggin, 500 ng/mL human recombinant R-spondin-1, 50 ng/mL murine recombinant EGF, 50 ng/mL Wnt3a (all from Peprotech) and 2.5 μmol/L GSK3i CHIR99021 (Cayman Chemical), and 10 μmol/L Rho kinase inhibitor Y-27632 (Tocris Bioscience). The organoids were cultivated in a water vapor–saturated atmosphere at 37°C and 5% CO_2_. The medium was changed every 3 to 4 days, and organoids were passaged once a week to remove dead cells and debris. To induce Cre recombination of floxed loci, organoids were treated with 3 μmol/L (Z)-4-hydroxytamoxifen (4-HT, Sigma-Aldrich) for 24 hours.

### Orthotopic transplantation

Col235 organoids originally derived from a male *Braf*^floxV600E/+^;*Trp53*^LSL-R172H/+^;*Apc*^flox/flox^;*Villin*::CreER^T2^ mouse were induced with 3.5 μmol/L 4-HT 2 days prior to transplantation. Before transplantation, organoids were incubated in TrypLE Express (Thermo Fisher Scientific) treatment at 37°C for 20 minutes and dissociated into single cells via thoroughly pipetting. Cells were counted and resuspended in culture medium (DMEM/F12 enriched with B-27 supplement, N-2 supplement, and 1 mmol/L N-acetylcysteine) supplemented with 10% Matrigel at a concentration of 100,000 cells/20 μL. Cells were maintained on ice until endoscopy-based transplantation to avoid Matrigel polymerization. To perform orthotopic transplantation into 8- to 12-week-old male and female syngeneic C57BL/6N recipients, a murine endoscopic system (COLOVIEW Mini Endoscopic System, Karl Storz) with a zero-degree optic (diameter 1.9 mm) was used. Prior to endoscopy, the mice were anesthetized with 2.5% (v/v) isoflurane (Piramal Critical Care), while keeping them under low-flow infusion (1.8% v/v) during the transplantation procedure. The mice were placed in a prone position on a heating pad, and a small amount of Bepanthen eye ointment (Bayer) was applied to protect their corneas. Gentle abdominal pressure was used to force stool pellets out of the distal rectum. The prepared single-cell suspensions were drawn up into a Hamilton syringe using a 22-gauge (G) transfer needle. The injection syringe was then attached to a 30 cm long 33 G injection needle. The endoscope was inserted carefully and under visual control up to approximately 4 cm into the colon (up to the first flexure), which was regulatory inflated by airflow. The endoscope was then slowly retracted to find suitable injection sites in the distal parts of the colon, which are characterized by exaltations or folds of the colonic lining. After reaching a suitable injection site, the 33 G injection needle was carefully introduced through the working channel of the endoscope into the colonic lumen. With a very gentle penetration of the epithelial layer, the tip of the needle was introduced into the submucosa at an approximately 20 to 30 degree angle relative to the colon wall and with its bevel (45 degrees) directed toward the lumen, and cells were injected. Successful implantation was indicated by bubble formation during injection of the cell suspension. A volume of 20 μL containing 10^5^ tumor cells was delivered for each injection site. Four injections were performed per mouse, covering the whole distal colon. The mice were weighed weekly, and tumor growth was monitored using endoscopy.

### Peritoneal lavage

Abdominal skin was removed without harming the peritoneum. Subsequently, 5 to 10 mL of cold DPBS was injected under inspection into the peritoneal cavity without injuring other organs. After 30 to 60 seconds, DPBS was gently aspirated with a syringe. The suspension was centrifuged at 200 rcf for 5 minutes. The supernatant was discarded. The cell pellet was then resuspended in 10 mL DMEM containing 10% fetal bovine serum (FBS), transferred to a TC25 cell culture flask, and incubated at 37°C with 5% CO_2_. The medium was changed every 2 to 3 days.

### Cell lines and their cultivation

The generation of the three NaJa cell lines is described in this work. Cells from passages 14 to 20 were used for most experiments. Simian virus 40 large T antigen immortalized murine embryonic fibroblasts; HEK293T and HT29 cells were taken from our institute stock (35, 36). The absence of *Mycoplasma* was confirmed by PCR using the service provided by Eurofins Genomics. Aliquots of HT29 stocks were authenticated by SNP analysis (Multiplexion) in June 2014 (37). All cell types were cultivated in DMEM (4.5 g/L glucose) supplemented with 10% FBS, 2 mmol/L L-glutamine, 10 mmol/L HEPES, penicillin (200 U/mL), and streptomycin (200 μg/mL), all purchased from PAN Biotech. Kinase inhibitors were obtained from Selleck Chemicals and dissolved in DMSO.

### Growth rate

NaJa cells were seeded in low density (20,000 cells per well) in six-well plates in triplicates for each time point. At the indicated time points, cells were trypsinized, stained with Trypan blue, and counted manually with a Neubauer chamber.

### Proliferation assay

NaJa cells were seeded in 96-well plates at 1,000 cells/well and grown for 14 days in the Incucyte Live-Cell Analysis System. The medium was changed every 2 to 3 days. Confluence was photographed every 2 hours and calculated by the Incucyte software.

### Colony formation assay

NaJa cells were counted, and 500 cells/well were seeded into a six-well culture plate with 2 mL medium. After 24 hours, the medium was removed and replaced with medium containing the indicated inhibitors or the correspondent amount of DMSO as a vehicle control. Each inhibitor concentration was applied in triplicates. The medium was changed every 2 days, and fresh inhibitors or DMSO were added at the indicated concentrations. After 10 days, the medium was discarded, and cells were fixed with 4% paraformaldehyde for 5 minutes and then dyed with 1 mL crystal violet (0.1% in ddH_2_O) per well. Wells were washed with 2 × 2 mL DPBS, rinsed with dH_2_O, and dried overnight. Pixel density was measured with Adobe Photoshop.

### Synergy assay

To calculate the synergy of two drugs, NaJa cells were seeded in 96-well plates with 3,000 cells per well. Using the Tecan D300e digital dispenser, inhibitors in logarithmic concentrations from low to high were dispensed singly and in combination. After 3 days, metabolic activity was determined using the Roche Cell Proliferation Kit II (XTT), and absorbance was measured 4 hours later at 590 nm (reference at 670 nm). Synergy was calculated with the help of SynergyFinder+ (38), and the LOEWE synergy score was calculated.

### Portal vein injection

NaJa cells were trypsinized, adjusted to 1 × 10^7^ cells per mL in PBS, and kept on ice until injection. For portal vein injection (PVI), mice were shaved and anesthetized with 2.5% (vol/vol) isoflurane (Baxter). Analgesia was provided via intraperitoneal injections of buprenorphine (0.1 mg/kg). Mice were positioned on their backs on a heating pad, with their legs secured using leukoplast tape. The isoflurane concentration was reduced to 1.8% (vol/vol). A 10- to 15-mm incision was made along the linea alba to open the abdomen. To expose the portal vein, the intestine was carefully exteriorized and continuously moistened with sterile PBS. NaJa cells (500,000 in 50 μL PBS) were then injected into the portal vein using a 32G needle. To prevent bleeding and minimize backflow of injected cells, the injection site was compressed for 6 minutes after injection. The intestine was then carefully repositioned into the body cavity, and the peritoneum and abdominal wall were closed with continuous sutures. Mice were provided drinking water supplemented with metamizole (5 mg/mL) for 3 days after surgery and monitored regularly.

### Immunofluorescence

The formalin-fixed, paraffin-embedded (FFPE) sections underwent standard processing and staining procedures using the following primary antibodies directed against E-cadherin (1:50, #610181, BD Laboratories; RRID: AB_397580) and Ki-67 (1:400, #9129, Cell Signaling Technology; RRID: AB_2687446). The fluorochrome-conjugated secondary antibodies Alexa Fluor 546 goat anti-mouse IgG and Alexa Fluor 488 goat anti-rabbit IgG (both 1:200, #A11003 and #A11008, Invitrogen; RRID: AB_2534071 and AB_143165) were used. The tissue was mounted with ProLong Gold Antifade with DAPI (Invitrogen) and cover slipped. Images were taken using the Axio Observer Z1 plus ApoTome 2 with an AxioCam by ZEISS and edited with the ZEN 3.0 blue edition software. For better visualization, a bold scale bar was manually fitted to the scale bar automatically generated by the microscope software.

### Immunohistochemistry

Slides with FFPE tissue sections were prepared and stained using standard protocols with the primary antibodies listed in Supplementary Table S1. Secondary antibodies were from the VECTASTAIN Elite ABC-HRP Kit, (rabbit IgG) or (rat IgG), (1:200, #PK-6101 and #PK-4004, Vector Laboratories). For visualization of the staining, slides were developed with diaminobenzidine (DAB) solution. Dependent on the antibody, DAB was applied for 5 to 15 minutes, and peroxidase reaction was stopped by transferring the slides into dH_2_O. Slides were counterstained with hematoxylin. The tissue was then mounted with Aquatex and cover slipped. Brightfield images were taken using the BZ-X810 microscope by Keyence and edited with the Keyence software. For better visualization, a bold scale bar was manually fitted to the scale bar automatically generated by the microscope software.

### Plasmids, viral transduction, and luciferase activity

The bicistronic retroviral vector pMIG encoding N-terminally HA-tagged human BRAF (or its point mutant V600E) together with enhanced green fluorescent protein was described previously (35). The pMIG/HA-BRAF^W450C^ mutant was generated by standard *PfuI*-mediated site-directed mutagenesis and the oligonucleotides W450Cfwd (5′-GAC​GGG​ACT​CGA​GTG​ATG​ATT​GTG​AGA​TTC​CTG​ATG​GGC​AGA​TTA​C-3′) and W450Crev (5′-GTA​ATC​TGC​CCA​TCA​GGA​ATC​TCA​CAA​TCA​TCA​CTC​GAG​TCC​CGT​C-3′). BRAF expression cassettes were confirmed by DNA sequencing. Retroviral pMIG particles were generated in Plat-E cells using the polyethyleneimine (PEI) procedure as described previously (35). The firefly luciferase expressing pLENTI_SV40_Lucif_Zeo vector was kindly provided by I.J. Frew. Lentiviral particles were produced in HEK293T cells using the ecotropic packaging system involving psPAX2 and pCMV-Eco (39). Transduced NaJa cells were selected in the presence of 300 μg/mL Zeocin (InvivoGen). Luciferase activity was determined by resuspending NaJa cells in lysis buffer (250 mmol/L KCl, 50 mmol/L HEPES, 0.1% IGEPAL, and 10% glycerol). Ten μl cleared lysate was mixed with 70 μL reaction buffer [25 mmol/L glycylglycine, 0.05 mmol/L luciferin, 2 mmol/L ATP (all from Sigma-Aldrich), and 10 mmol/L MgCl_2_]. Measurement was taken on a Tecan Infinite M200, and photon counts were normalized to protein concentration.

### Western blot

To generate total cell lysates (TCL), cells were lysed with modified RIPA buffer [50 mmol/L Tris/HCl, pH 7.5; 1% NP-40/IGEPAL; 0.5% sodium deoxycholate, 0.1% sodium dodecylsulfate, 137 mmol/L sodium chloride; 1% glycerine; 1 mmol/L sodium orthovanadate; 0.5 mmol/L EDTA; 0.01 mg/mL leupeptin, 0.1 mg/mL aprotinin, and 1 mmol/L 4-(2-aminoethyl)benzenesulfonyl fluoride hydrochloride]. Protein concentration was determined using the Pierce BCA Protein Assay Kit (Thermo Fisher Scientific). Lysates were separated on a 10% polyacrylamide gel and then transferred to a 0.45 μm polyvinylidene fluoride membrane (Thermo Fisher Scientific) by Western blotting technique (100 V/75 minutes). Unspecific binding of antibodies was blocked by incubating the membrane in 2.5% BSA in PBST for a minimum of 30 minutes. Standard Western blot analysis was performed using the primary antibodies listed in Supplementary Table S1. The membrane was then incubated at room temperature for 1 hour with horseradish peroxidase–conjugated anti-mouse or anti-rabbit secondary antibodies (800 μg/mL, Thermo Fisher Scientific) in 3% skim milk in PBST. The proteins were visualized with the enhanced chemiluminescence Western blotting substrate using the FUSION-SL Advanced System. In case, lysate aliquots were run on different gels, equal loading/transfer was confirmed for each gel/PVDF membrane by gel-specific loading controls.

### Protein arrays

To conduct a phospho-RTK array, NaJa cells were treated for 5 days with the indicated inhibitors, before culture dishes were washed with PBS and covered with lysis buffer. Cells were removed from the dish with a cell scraper and transferred to an Eppendorf tube. After a 15-minute incubation time on ice, samples were centrifuged at 16,100 rcf and 4°C for 10 minutes. The supernatant was collected, and the protein concentration was determined using the Pierce BCA Protein Assay Kit. From each sample, the recommended amounts (250 μg) of protein were used for the Proteome Profiler Mouse Phospho-RTK Array Kit (ARY0141, R&D systems). To conduct a cytokine array, NaJa cells were treated for 5 days with the indicated inhibitors before the supernatant was harvested. The cells were lysed, and the protein concentration was determined using the Pierce BCA Protein Assay Kit. From each sample, the supernatant was normalized to the correspondent protein amount before they were used for the Proteome Profiler Mouse XL Cytokine Array (ARY028, R&D systems). The arrays were visualized using a Fusion Solo chemiluminescence reader (Vilber Lourmat). All arrays of one experiment and one cell line were exposed simultaneously. For proper validation of the entire array, three adequate exposure times (short, middle, and long) were chosen per cell line to properly quantify the log_2_ fold change (FC) of highly abundant cytokines/RTKs but also the less abundant cytokines/RTKs. Pixel density was quantified using HLImage++ by Western Vision Software.

### *In vitro* kinase assay

Two million HEK293T cells were plated onto 10-cm plates. On the next day, cells were transfected with 8 μg of the indicated pMIG/HA-BRAF vectors using the PEI protocol (35) and lysed with 1 mL modified RIPA buffer 2 days later. One hundred μL of cleared TCL were removed, mixed with Laemmli buffer, denatured by boiling at 98°C for 5 minutes, and used for TCL analysis. Subsequently, 0.4 μg rat anti-HA antibody 3F10 (Roche) was added to 800 μL TCL for 1 hour, followed by incubation with Protein G-Sepharose beads (GE Healthcare) at 4° under agitation and for 2 hours. Beads were washed six times with 1 mL RIPA buffer, followed by five washes with 1 mL Kinase assay buffer (KAB; 20 mmol/L 4-morpholinepropanesulfonic acid, pH 7.2; 25 mmol/L β-glycerol phosphate; 5 mmol/L EGTA, 1 mmol/L dithiothreitol, and 1 mmol/L sodium orthovanadate). The beads were then resuspended in 100 μL KAB, and 10 μL of this suspension were mixed with 1 μg recombinant GST-MEK1 (Upstate) and 5 mmol/L ATP in 38 μL KAB. The i*n vitro* kinase assay (IVK) reaction was shaken at 30°C for 30 minutes and immediately stopped by the addition of 5 × Laemmli buffer and boiling at 98°C for 5 minutes. The IVK reactions were size-separated on 10% SDS–PAGE, and phosphorylated GST-MEK1 and HA-tagged BRAF proteins were detected with phospho-MEK and anti-HA (3F10; Roche) antibodies, respectively.

### RNA sequencing

For the RNA sequencing (RNA-seq) of organoids, organoid cultures of two donor mice per genotype were induced with 3.5 μmol/L 4-HT for 24 hours. RNA was isolated 3 days later as previously described (23) using the Universal RNA Purification Kit (EURx, E3598). For the RNA-seq of NaJa cells, cells were plated at the same time and treated continuously for either 1 day or 5 days with 0.5 μmol/L encorafenib before RNA isolation. Consistent confluence was confirmed before harvest. RNA quality was checked with the Agilent 2100 Bioanalyzer. Following quality control, reads were aligned to the mouse GRCm38/mm10 reference genome using the STAR aligner (40). Normalization and differential expression analysis were performed with the Bioconductor package limma (41, 42). Only genes with a higher count across all samples, i.e., the row sum of a gene higher than two in a gene by sample matrix, were kept. The normalization was calculated with the Trimmed Mean of M-values method considering the library size. The significance cutoff (adjusted *P* value) for the genes was <0.01, calculated by the Benjamini–Hochberg method. Enrichment analysis was performed using the Bioconductor package GAGE (43) with signaling pathways from Gene Ontology (44). Pathways were considered significant with an adjusted *P* value < 0.1 (Benjamini–Hochberg). Gene set variation analysis (GSVA; ref. 45) was done using the Bioconductor package GSVA with the Hallmark gene sets from the MSigDB (46).

### Whole-exome sequencing

To extract genomic DNA of NaJa cells and the donor organoids, the QiaAmp DNA Micro Kit (QIAGEN, cat. #56304) was used according to the manufacturer’s instructions. The Agilent SureSelect XT Mouse All Exon Capture Kit was used for library preparation, and the samples were subsequently sequenced on the Illumina NovaSeq 6000 platform with a read length of 100 base pairs (bp). Sequencing data were processed with Trimmomatic (47) to remove sequencing adapters and low-quality reads. The processed reads were then mapped to the GRCm38/mm10 genome assembly using the Burrows–Wheeler Aligner (48). Variants were identified using Mutect2 from the Genome Analysis Toolkit (49). The variants detected included single-nucleotide variants as well as insertions and deletions up to 30 bp in length, which were further annotated using ANNOVAR (50).

### Statistical analysis

GraphPad Prism 10.3.1 software was used for data analysis. Unless stated otherwise, data are shown as the mean ± SD. Statistical significance was determined by ordinary one-way ANOVA with Dunnett multiple comparisons test if all samples were compared with a single control or Tukey multiple comparisons test if all samples were compared with each other. Nonparametric data were analyzed using the Kruskal–Wallis test followed by Dunn multiple comparisons test. *P* values less than 0.05 were considered statistically significant and depicted as *, *P* < 0.05; **, *P* < 0.01; ***, *P* < 0.001; and ****, *P* < 0.0001.

## Results

### BPAC organoids show aggressive growth behavior and drug resistance

Although BRAF^V600E^ mutant colorectal cancers were originally considered WNT-independent, recent analyses on the co-mutation spectrum in human colorectal cancers as well as functional studies in mice (or their organoids) demonstrated that both pathways must cooperate in tumor progression (23–25, 28, 29). Of note, Fennell and colleagues (24) reported that 21% of MSS^+^ colorectal cancers contained *BRAF*^*V600E*^ and *APC* alterations and represent a highly aggressive subset associated with poor survival. More recent studies even reported frequencies between 29% and 35% for this genotype (16, 29, 51). Mechanistically, this could be explained by our previous study demonstrating that biallelic *Apc* deficiency rescues murine colon organoids from BRAF^V600E^-induced disintegration of their ISC niche and allows their continuous but MEK-dependent proliferation *in vitro* (23). This prompted us to investigate this colorectal cancer subtype in more detail using organoid models *in vitro* and upon retransplantation *in vivo* (Supplementary Fig. S1–S3). Therefore, we generated colon organoids from C57BL/6N mice harboring the following conditional *knock-in*/*knockout* alleles, either singly or in combination: *Braf*^*floxV600E*^ (B), *Trp53*^*LSL-R172H*^ (P), and *Apc*^*flox*^ (A) (Supplementary Fig. S1A). The donors also carried the *Villin::CreER*^*T2*^ transgene (C) allowing the inducible expression of mutant BRAF^V600E^ and p53^R172H^ upon 4-HT–mediated Cre activation. Our previous study provided an in-depth analysis of the effects of BRAF^V600E^ and p53^R172H^ in small and large intestinal organoids and a first hint at their cooperation with APC loss (23). In this study, we extend these analyses by showing that *Apc* deletion, either by itself or in combination with p53^R172H^ induction, had no discernible effect on organoids, which displayed the typical elongated crypt areas (Supplementary Fig. S1B). BAC and BPAC organoids, however, displayed numerous crypts and developed a shorter, rounder form upon 4-HT exposure. Staining for the S-phase marker Ki-67 confirmed that proliferation was restricted to crypts in vehicle control–treated [and hence “wild-type” (WT)] BPAC organoids, whereas 4-HT induced proliferation throughout the entire organoid. As *Apc* deficiency prevented organoid disintegration that is normally observed upon the induction of oncogenic BRAF in small or large intestinal organoids (21, 23, 27, 28), we were particularly interested to dissect the relative contributions of BRAF^V600E^, mutant p53, and *Apc* deficiency on organoid transcriptomes by bulk RNA-seq. ISC markers and WNT targets were significantly upregulated in BAC and BPAC organoids, which could explain the survival and growth factor independent proliferation of the organoids (Supplementary Fig. S1C and S1D). Commensurate with earlier findings (52), *Apc* deficiency induced transcripts of *Sox9*, a known WNT target and transcription factor blocking intestinal differentiation. Interestingly, transcripts for *Lgr5*, the ISC niche–defining marker (53), were particularly abundant in *Apc*-deficient organoids with concomitant BRAF^V600E^ expression. Furthermore, epithelial differentiation markers were downregulated across all samples, with a more pronounced effect observed for BRAF^V600E^-expressing organoids (Supplementary Fig. S1E). This was mirrored by the reduced E-cadherin staining in the 4-HT–treated BPAC organoids compared with their uninduced controls (Supplementary Fig. S1B). When looking at ERK target genes, we observed the expected, stronger change in the expression in BAC and BPAC organoids (Supplementary Fig. S1F).

Next, we tested whether 4-HT –recombined BPAC organoids responded to clinically relevant targeted therapy compounds (Supplementary Fig. S2A and S2B). To this end and inspired by the BEACON protocol (12), we chose encorafenib, the MEKi trametinib, and the pan-EGFR/HER family kinase inhibitor afatinib. Based on previous studies (54–56), we applied clinically meaningful concentrations (0.1 μmol/L for afatinib, 1 μmol/L for encorafenib, and 25 nmol/L for trametinib) but also lower and higher concentrations to gauge drug responses more carefully. We decided to explore trametinib in more detail, as it exhibits, compared with binimetinib, superior efficacy upon combination with encorafenib in human melanoma cell lines (57). Afatinib was chosen for two reasons. First, cetuximab does not recognize the murine EGFR (58), and second, afatinib would block the activity of other EGFR/HER family members that are often upregulated in human colorectal cancer cell lines upon ERK pathway inhibition and could therefore prolong therapeutic responses (7, 59). Indeed, 4-HT–induced BPAC organoids were resistant to either encorafenib or afatinib single treatment but remained sensitive to their combination as well as to trametinib. This indicates that the BEACON concept can be recapitulated in BPAC organoids with clinically relevant kinase inhibitors.

### Orthotopically transplanted BPAC organoids generate metastatic tumors in syngeneic immunocompetent mice and give rise to new cell line models

**Figure 1.**
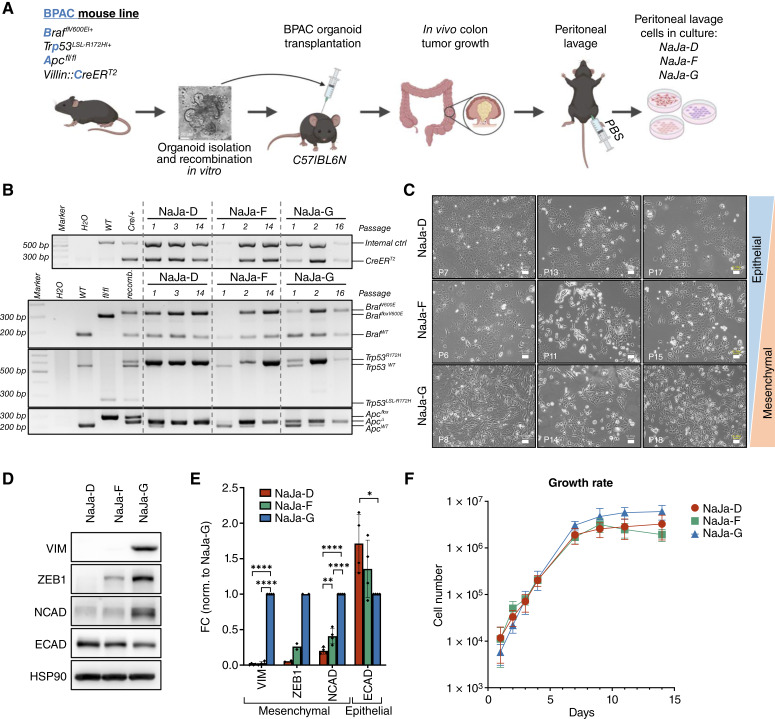
NaJa cells share the same driver mutations but differ in morphology and EMT marker expression. **A,** Flow chart of NaJa cell generation. Colon organoids were isolated from BPAC mice, expanded *in vitro* and exposed to 4-HT to induce Cre-mediated recombination of the floxed *Braf*, *Trp53*, and *Apc* loci prior to orthotopic injection into the colon of syngeneic C57BL/6N mice. NaJa cells were isolated from three individual recipients by peritoneal lavage. **B,** PCRs confirming the presence of the recombined *Apc*^Δ^, *Braf*^V600E^, and *Trp53*^R172H^ alleles as well as the presence of the *Villin*::CreER^T2^ transgene over different passages. As controls, genomic WT DNA, genomic DNA containing the *lox*P sites (fl/fl), and recombined (recomb.) genomic organoid DNA was used. The WT band indicated in the CreER^T2^ amplifying PCR (top) represents an internal control band validating template DNA quality. **C,** Brightfield images showing the distinct morphologies of the NaJa cell lines over three different passages. Scale bar, 50 μm. **D,** Western blot detections of the EMT markers vimentin (VIM), ZEB1, N-cadherin (NCAD), and E-cadherin (ECAD) with HSP90 serving as a loading control. **E,** Quantification of the data shown in (**D**) and three additional biological replicates. Data are presented as the mean ± SD and were normalized to NaJa-G. Statistical significance was calculated using one-way ANOVA and Dunnett multiple comparisons test for each protein individually. *, *P* < 0.05; **, *P* < 0.01; ****, *P* < 0.0001. **F,** Growth rate comparison between the NaJa cell lines. Shown is the mean cell number from two biological replicates. Technical triplicates were counted at each time point. [**A,** Created by J. Traichel in BioRender. Brummer, T. (2025) https://BioRender.com/94gtski.]

With the aim of creating an immunocompetent *in vivo* model for BRAF^V600E^-driven colorectal cancer, we next explored the tumorigenic potential of 4-HT–treated BPAC organoids by their orthotopic transplantation into syngeneic and immunocompetent C57BL/6N mice (Fig. 1A). We observed heterogeneous tumor growth within 63 to 149 days in 8 of 18 recipients (44%; Supplementary Fig. S3A). The resulting tumors varied in size and carried Cre-recombined *Apc* alleles, thereby confirming their organoid origin (Supplementary Fig. S3B and S3C). Furthermore, most primary tumors expressed the homeobox transcription factor CDX2, which controls multiple effector functions of differentiated intestinal epithelium (60). Moreover, CDX2-positive metastases were observed in the liver and prostate (Supplementary Fig. S3A and S3C).

As human BRAF^V600E^-driven colorectal cancers frequently develop peritoneal metastases (5, 61), we cultured peritoneal lavages from all hosts to monitor for outgrowing cells derived from the donor organoid. After culturing the lavages in conventional tissue culture medium without any added growth factors, we indeed found cells showing Cre-mediated recombination of all three BPAC alleles in three of eight peritoneal lavages, presumably representing metastatic cells (Supplementary Fig. S4A). The cells from the peritoneal lavages of recipients D, F, and G were grown for several passages during which host cells were lost as reflected by the disappearance of the WT *Apc* allele–derived PCR amplicon (Fig. 1B). In contrast to the donor organoid (Col235), all outgrowing cells demonstrated a loss of heterozygosity in the *Trp53* allele, indicated by the disappearance of the WT allele-derived amplicon at higher passage numbers and the increased frequency of *Trp53*^*R172H*^ allele in our whole-exome sequencing (WES) analysis (Fig. 1B; Supplementary Fig. S4B). These data further underscore the absence of host cells, e.g., fibroblasts, at higher passage numbers and suggest that the *Trp53*^*R172H*^ allele is subject to positive selection.

Importantly, these cancer cell isolates could be easily propagated as stable cell lines, which were henceforth named IMMZ-NaJa-D, -NaJa-F, and -NaJa-G according to the acronym of our home institute and the first names of the experimenters. As there is no published murine cell line model for MSS^+^ BRAF^V600E^-driven colorectal cancer to date, these isolates represent a unique opportunity to establish a reliable immunocompetent model, prompting us to shift our focus toward their systematic characterization. The so-called NaJa cells display distinct phenotypes, ranging from the epithelial-like NaJa-D to the mesenchymal-like NaJa-G cells and NaJa-F cells showing intermediate features (Fig. 1C). These cellular phenotypes correlated with various epithelial–mesenchymal transition (EMT) markers, showing that the mesenchymal NaJa-G cells express the highest level of the EMT master regulator ZEB1 and the mesenchymal markers vimentin and N-cadherin (Fig. 1D and E; ref. 62). In contrast, NaJa-D cells expressed the highest level of the epithelial marker E-cadherin but hardly detectable to low levels of vimentin, ZEB1, and N-cadherin. Despite these differences, all NaJa lines displayed similar proliferation rates (Fig. 1F; Supplementary Fig. S4C), and WES revealed no obvious mutations that could explain the different phenotypes (Supplementary Fig. S5; Supplementary Table S2). WES also revealed no mutations in microsatellite stability markers (*Bat24*, *Bat26*, *Bat30*, *Bat37*, and *Bat64*) and mismatch repair genes (*Mlh1*, *Msh2*, *Msh6*, and *Pms2*), which classifies all three lines as proficient mismatch repair/MSS.

### NaJa cells respond to clinically relevant inhibitors

**Figure 2.**
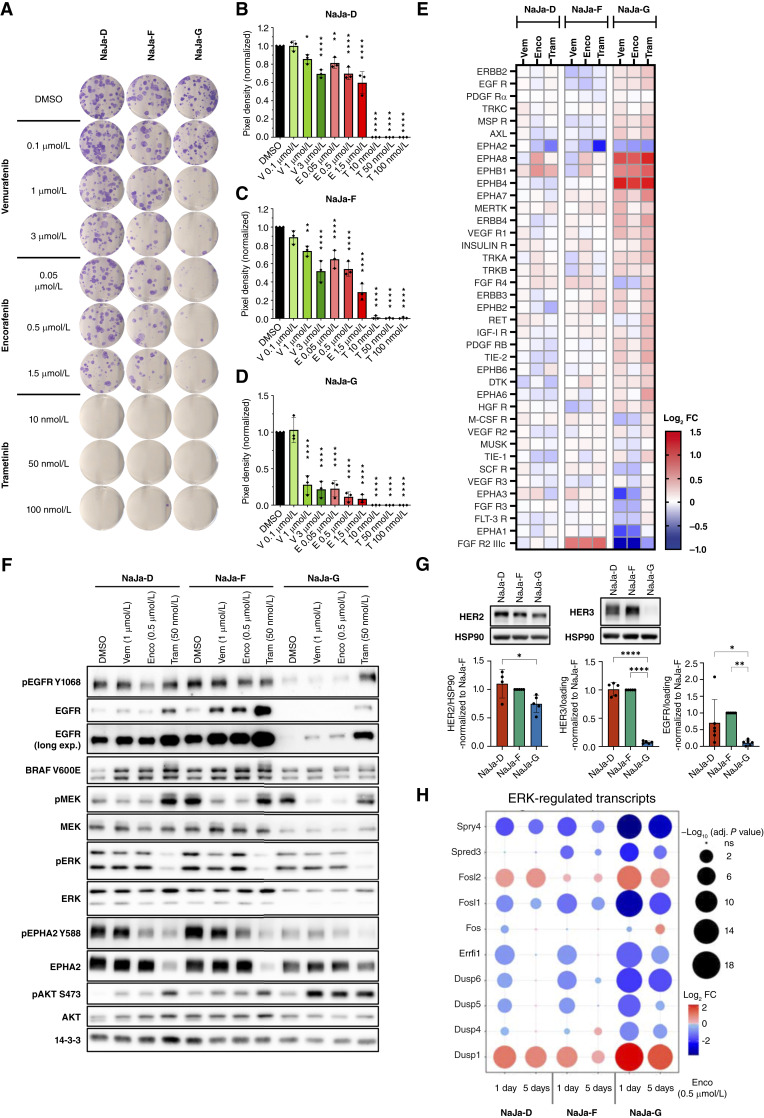
NaJa cells differ in their response to drug treatment. **A,** Colony formation assay of NaJa cells treated with vemurafenib (V), encorafenib (E), and trametinib (T) at the indicated concentrations for 10 days. Shown is a representative result from three biological replicates. **B–D,** Quantification of the data shown in **A**. Data are presented as the mean ± SD and were normalized to the DMSO control (*n* = 3). Statistical significance was calculated using one-way ANOVA and Dunnett multiple comparisons test. *, *P* < 0.05; **, *P* < 0.01; ****, *P* < 0.0001. **E,** Phospho-RTK array of NaJa cells treated with 1 μmol/L vemurafenib (Vem), 0.5 μmol/L encorafenib (Enco), or 50 nmol/L trametinib (Tram) for 5 days. Shown are the log_2_ FCs of treated cells versus DMSO control. Array membranes and cell line specific heatmaps are displayed in Supplementary Fig. S7. **F,** Western blot showing the effect of a 5-day treatment of the NaJa cells on various signaling pathways. **G,** Western blot and quantification of HER2 and HER3 (*n* = 4). Basal EGFR expression was quantified based on data in (**F**), and five additional biological replicates normalized to the internal loading control. Data are presented as the mean ± SD and were normalized to NaJa-F for comparison. Statistical significance was calculated using one-way ANOVA and Tukey multiple comparisons test. *, *P* < 0.05; **, *P* < 0.01; ****, *P* < 0.0001. **H,** Bulk RNA-seq of NaJa cells treated with 0.5 μmol/L encorafenib for 1 day or 5 days showing upregulation (red) or downregulation (blue) of ERK target genes and the adjusted *P* value (bubble size).

Next, we tested the response of the three NaJa lines toward vemurafenib, encorafenib, as well as the MEKi trametinib, all which are regularly used to treat BRAF^V600E^-driven cancers (3). Although BRAFis are often combined with MEKis and in colorectal cancer even with EGFR-targeting antibodies (12), we first evaluated the responses to single treatments to obtain better insights into the mechanism underlying the observed drug responses. Surprisingly, the NaJa lines differently reacted toward the targeted therapy compounds, despite their common origin and highly similar mutational background (Supplementary Fig. S5). Although trametinib almost abolished colony formation in all three lines, vemurafenib and encorafenib induced varying responses, with NaJa-D showing major drug resistance, NaJa-G being very sensitive, and NaJa-F showing an intermediate response (Fig. 2A–D). The higher concentrations of vemurafenib were chosen as this BRAFi has a lower affinity than encorafenib (11, 63). We also compared trametinib with the BEACON MEKi, binimetinib, in a titration experiment, showing higher potency of trametinib at clinically relevant concentrations (Supplementary Fig. S6).

As ERK pathway inhibition upregulates various RTKs in human colorectal cancer cell lines (7) and to obtain deeper insights into the differential drug responses of the NaJa cells, we performed a phospho-RTK array and Western blot analyses (Fig. 2E and F; Supplementary Fig. S7A and S7B). Again, there were marked differences between the cell lines, but all had in common that the amount of total EGFR was upregulated by BRAFis and MEKis (Fig. 2F). NaJa-G cells further demonstrated a pronounced increase in treatment-induced phosphorylation of EGFR and HER2/ERBB2, as detected by phospho-RTK array. This was corroborated by Western blot analysis confirming increased trametinib-induced phosphorylation of EGFR at Y1068 (Fig. 2F). Furthermore, ERK activation as well as the expression and phosphorylation of EPHA2, a known transcriptional ERK target (64), were suppressed by trametinib (Fig. 2E and F). This mirrors the strong MEKi sensitivity observed in the colony formation assay. Among the RTKs, EPHA2 showed one of the strongest reductions in phosphorylation, whereas other ephrin receptors, such as EPHA8 or EPHB1, became strongly phosphorylated. Interestingly, AKT was strongly phosphorylated upon treatment in all three lines, with NaJa-D showing the lowest baseline level of AKT phosphorylation. Again, NaJa-G displayed the strongest increase in AKT phosphorylation at the mTORC2 target site S473, which is consistent with the strong increase in RTK phosphorylation that is expected to fuel PI3K/mTOR signaling (65). Nevertheless, this increase in AKT activity could not confer BRAF/MEK inhibitor resistance (Fig. 2A). In summary, these data highlight that the three NaJa lines differ in their signaling ground states and drug responses.

To further investigate the different ground states of the NaJa cells, we compared their baseline transcriptomes using bulk RNA-seq (Supplementary Fig. S8A). Among many notable cell line–specific differences, this analysis confirmed the elevated PI3K–AKT–MTOR signaling pathway in NaJa-F and -G compared with NaJa-D. AKT activation was also confirmed by Western blotting revealing significantly increased levels of S473 phosphorylation (Fig. 2F; Supplementary Fig. S8B). Commensurate with the differences in EMT marker expression (Fig. 1D and E), the most prevalent induction of EMT-associated transcripts was observed in NaJa-G. Moreover, the RNA-seq analysis revealed additional cell line–specific differences at baseline, such as the more abundant expression of transcripts encoding EMT-associated RTKs like Pdgfra, Met, and the tumor-associated macrophage [(TAM) Tyro3, Axl, and Mertk)] family (66–69) and the reduction in EGFR family (Egfr, Erbb2, and Erbb3) expression in NaJa-G (Supplementary Fig. S8C). The latter finding was also confirmed at the protein level (Fig. 2G).

Next, we monitored the BRAFi-mediated effects on the transcriptomes of the three NaJa lines (Fig. 2H). Following a 1- or 5-day treatment with encorafenib, ERK-regulated transcripts were most strongly downregulated in the most sensitive line, NaJa-G. Although all lines initially responded and downregulated most ERK targets, this effect waned off after 5 days of continuous encorafenib treatment, particularly in NaJa-D and NaJa-F. This agrees with Fig. 2F showing that ERK phosphorylation was unaffected by 5 days of encorafenib treatment and suggests the emergence of a potential resistance mechanism. In line with the colony-forming assays, RNA-seq revealed the encorafenib-upregulated transcripts for the antiproliferation factor BTG2 and for proapoptotic effectors such as decorin and the BH3 only proteins BIK, NOXA (*Pmaip*1), BID, and BMF in all three cell lines (Supplementary Fig. S9A). Interestingly, NaJa-G, which exhibited the highest encorafenib sensitivity, displayed an induction and reduction in the transcripts for the proapoptotic BH3 only protein BIM (*Bcl2l*11) and the anti-apoptotic BCL2 family member BCL-W (*Bcl2l2*), respectively. High expression levels of the latter have been linked to aggressive human colorectal cancers (70). Interestingly, encorafenib also induced transcripts encoding inflammatory proteins such as IL18 and IL33 and their processing enzyme CASP4 (Supplementary Fig. S9A; Supplementary Table S3).

### NaJa cells require BEACON targets for proliferation

**Figure 3.**
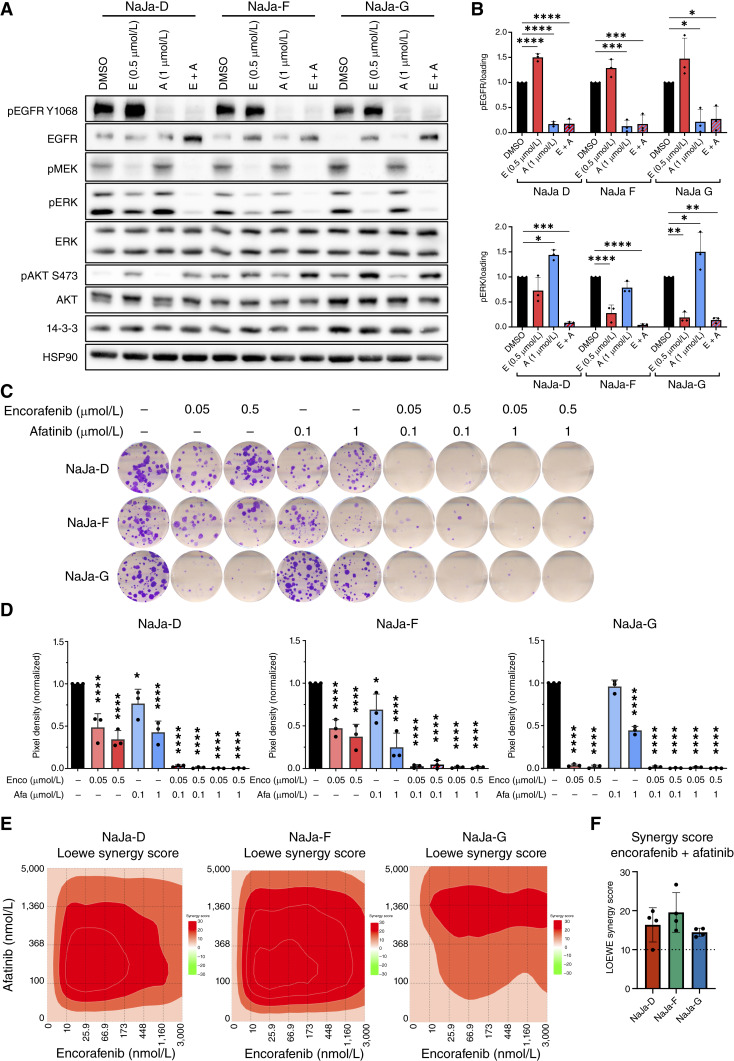
Simultaneous inhibition of BRAF^V600E^ and the HER family receptors leads to a reduction in signaling and viability in the NaJa cell lines. **A,** Representative Western blot (WB) analysis of NaJa cell lines, treated with 0.5 μmol/L encorafenib (E) and 1 μmol/L afatinib (A), either alone or in combination (E + A), for 24 hours. HSP90 and 14-3-3 serve as loading controls. **B,** Quantification of WB analysis of pEGFR and pERK normalized to the internal loading control (*n* = 3 biologically independent experiments). Data are presented as the mean ± SD. Statistical significance was calculated using one-way ANOVA and Dunnett multiple comparisons test for each cell line individually. **C,** Colony forming assays of NaJa cell lines treated for 10 days with encorafenib and afatinib, either alone or in combination. Shown is a representative result from three independent biological replicates. **D,** Quantification of the data shown in **C**. Data are presented as the mean ± SD and were normalized to the DMSO control (*n* = 3). Statistical significance was calculated using one-way ANOVA and Dunnett multiple comparisons test. *, *P* < 0.05; **, *P* < 0.01; ***, *P* < 0.001; ****, *P* < 0.0001. **E,** Representative surface plots illustrating the synergistic effects of encorafenib/afatinib cotreatment observed in XTT assays. **F,** Quantification of the synergy scores, as calculated by LOEWE (*n* = 4). A synergy score of >10 indicates a synergistic effect between two drugs. Data are presented as the mean ± SD.

Given the different RTK signaling dynamics, in particular of EGFR/HER family members (Fig. 2E and F), and to explore the feasibility of inhibiting the BEACON trial targets in our cell lines, we combined encorafenib with afatinib (Fig. 3A). Phosphorylation as well as total expression of the EGFR became upregulated 24 hours after encorafenib treatment (Fig. 3A and B). The three lines reside in different ground states with the EGFR well detectable in NaJa-D/F prior to encorafenib exposure, although this RTK only becomes readily detectable in NaJa-G following treatment. Importantly, afatinib suppressed both basal and encorafenib-induced EGFR phosphorylation. ERK phosphorylation was more prominently inhibited with the combination of both compounds, as encorafenib alone insufficiently inhibited ERK phosphorylation in NaJa-D (Fig. 3A and B). By comparing the ERK phosphorylation differentials of DMSO and encorafenib single-treated cells after 1 (Fig. 3A) and 5 days (Fig. 2F), we noticed a rebound effect for this BRAFi. This observation ties in with the RNA-seq data showing a slight relief of encorafenib-mediated suppression of ERK target genes at day 5 (Fig. 2G). In line with Fig. 2, AKT phosphorylation at S473 was upregulated after 24 hours of encorafenib treatment, but interestingly this signaling event was independent of the EGFR phosphorylation status.

The encorafenib/afatinib combination efficiently suppressed colony formation in all lines by at least 95%, whereas either of the drugs alone was insufficient to inhibit the growth of NaJa-D and -F (Fig. 3C and D). This effect and the increased sensitivity of NaJa-G were also reflected in a drug washout experiment (Supplementary Fig. S9B and S9C). By performing XTT assays after 3 days of treatment, we observed a synergistic effect of the encorafenib plus afatinib combination in all three cell lines (Fig. 3E and F). Thus, the NaJa cells represent a promising platform for preclinical studies, as they display diverse responses to the BEACON triplet regimen thereby reflecting the variability seen in patients.

Vertical pathway inhibition by combining BRAFis and MEKis typically yields more durable responses in various tumors. Early clinical data suggested that adding a MEKi to the encorafenib/cetuximab doublet offers slight survival benefits (12, 15, 71). MEKis, however, often cause dose-limiting toxicities, raising questions about their necessity. Their main rationale is to prevent ERK pathway reactivation, as BRAF^V600E^-selective inhibitors cannot block RAF dimers and may even cause paradoxical MEK/ERK signaling in RTK/RAS-activated cells (3, 6). This has driven the development of dimer-selective RAFis to overcome these limitations (63). Therefore, we tested one of these clinically trialed compounds, exarafenib (72). Following a titration experiment in which we determined various exarafenib doses for a reduction in pMEK/pERK levels by at least 90% (Supplementary Fig. S10A), we chose 0.05 and 0.5 μmol/L, with the latter showing efficacy against various human cancer cell lines with distinct BRAF alterations and still being within a range reported in human and murine plasma (72–74). Interestingly, exarafenib seems more potent than encorafenib, as it effectively inhibited MEK/ERK phosphorylation and colony formation as a single agent (Supplementary Fig. S10B–S10E). Moreover, when calculating synergy scores, exarafenib did not show a synergistic but rather an additive effect with afatinib (Supplementary Fig. S10C). This suggests that EGFR blockade might be less critical in the context of exarafenib. Indeed, we did not observe an upregulation of EGFR phosphorylation in any of the cell lines after 24 hours of treatment (Supplementary Fig. S10E). This represents an interesting observation, in particular as an *in vitro* kinome screen excluded the WT EGFR as an exarafenib target, although the activity of some activated EGFR mutants was reduced by 10% to 20% (72). The efficacy of exarafenib and its contrasting behavior to encorafenib invite follow-up studies in murine and human preclinical colorectal cancer models and might help to understand the results from an ongoing basket trial including patients with colorectal cancer (1, 75).

### NaJa cells successfully establish tumors *in vivo*

**Figure 4.**
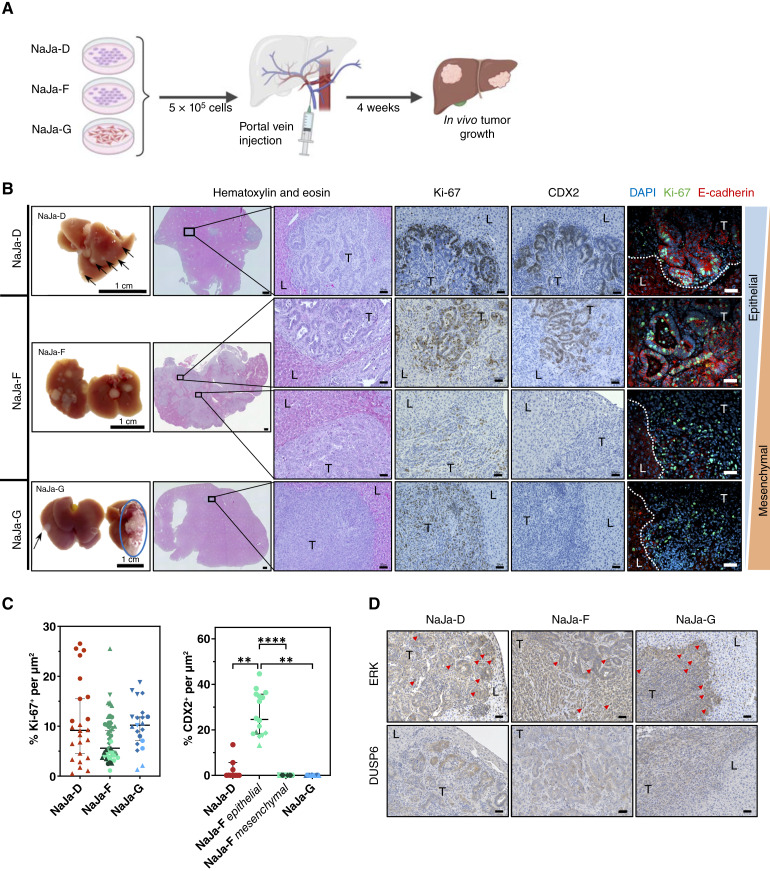
All NaJa cell lines successfully engraft into syngeneic immunocompetent C57BL/6N mice and recapitulate their individual 2D morphologies *in vivo*. **A,** Schematic overview of the PVI of NaJa cells (*n* = 2 NaJa-D, *n* = 10 NaJa-F, and *n* = 3 NaJa-G). **B,** Left, livers showing metastatic nodules 4 weeks after injection (black arrows) and invasive growth in the pancreatic tissue (blue circles). Scale bar, 1 cm. Hematoxylin and eosin: Histology showing that all NaJa cell lines establish tumors (pale blue) in the liver parenchyma (pink) with a differentiation state matching their epithelial or mesenchymal *in vitro* morphology. Right, IHC and immunofluorescence (IF) staining of proliferation marker Ki-67 (left) and intestinal differentiation markers CDX2 (middle) and E-cadherin (right). L, liver; T, tumor. Scale bar (overview), 500 μm, scale bar (zoom, IHC, and IF), 50 μm. **C,** Quantification of Ki-67 and CDX2 IHC staining in NaJa cell–induced liver metastases. Each dot represents an individual tumor; different shapes indicate individual tissue sections, and colors denote individual mice. Data are shown as the median ±95% confidence interval (CI).Statistical significance was assessed using the Kruskal–Wallis test followed by Dunn multiple comparison test. **, *P* < 0.01; ****, *P* < 0.0001. **D,** IHC staining of ERK and its downstream target DUSP6. Red arrows indicate examples of nuclear ERK staining. L, liver; T, tumor. Scale bar, 50 μm. [**A,** Created by J. Traichel in BioRender. Brummer, T. (2025) https://BioRender.com/8ogg64c.]

Next, we determined whether NaJa cells could be retransplanted into syngeneic C57BL/6N mice and therefore serve as a preclinical and more reliable *in vivo* model for BRAF^V600E^-driven metastatic colorectal cancer than organoids. As we aimed to model colorectal cancer as a systemic disease, we chose a pseudometastatic *in vivo* model mimicking a common route of metastatic colorectal cancer, hematogenic dissemination. Therefore, NaJa cells were injected into the portal vein of immunocompetent C57BL/6N mice to investigate their tumorigenic potential in the liver (Fig. 4A). As shown in Fig. 4B, all three lines successfully engrafted in all transplanted mice (*n* = 2 NaJa-D, *n* = 10 NaJa-F, and *n* = 3 NaJa-G) 4 weeks after inoculation, and macroscopic tumors were even detectable in some mice 2 weeks after injection. Aggressive tumor growth was observed in the liver as well as in or on nearby organs such as the pancreas, peritoneum, intestine, and kidney (Supplementary Fig. S11). Remarkably, all allografts displayed morphologic features matching the differentiation characteristics observed by microscopy, Western blotting, and RNA-seq *in vitro* (Fig. 1C–E; Supplementary Fig. S8A and S8C). NaJa-D tumors seemed relatively well differentiated with “colon crypt–like” structures and prominent expression of E-cadherin and CDX2. In contrast, tumors arising from NaJa-G cells displayed a highly mesenchymal and dedifferentiated histology, lacking CDX2 and E-cadherin. Strikingly, NaJa-F–derived tumors presented as a mosaic of epithelial “colon-like” CDX2-positive lesions and mesenchymal CDX2-negative lesions. In addition, tumors derived from all three NaJa lines showed a high number of Ki-67^+^ proliferating cells (Fig. 4B and C). ERK pathway activation was maintained in the induced tumors, as indicated by nuclear ERK staining [Fig. 4D (red arrows)] and increased expression of its downstream target DUSP6 (Fig. 4D). Overall, the data showed that NaJa cells reliably induced tumor formation in immunocompetent mice while maintaining their *in vitro* phenotypes, making them a versatile and more reliable model for further preclinical studies than our previously presented organoid approaches.

Bioluminescence imaging using transgenes for fluorescent or light emitting proteins represents an important noninvasive technique to monitor allograft or xenograft longitudinally, in particular under treatment (76). Some of these transgenic marker proteins, however, can be highly immunogenic and trigger rejection of transplanted cells within fully immunocompetent syngeneic hosts (77). To rule out this potential caveat for future studies, we transduced the cells with an expression vector for firefly luciferase. Despite their prominent luciferase expression, the tumorigenic potential of NaJa cells was not affected, making them a useful tool for future longitudinal studies modeling drug responses (Supplementary Fig. S12A–S12C).

### Characterization of immunomodulatory features in NaJa cells and their tumors

**Figure 5.**
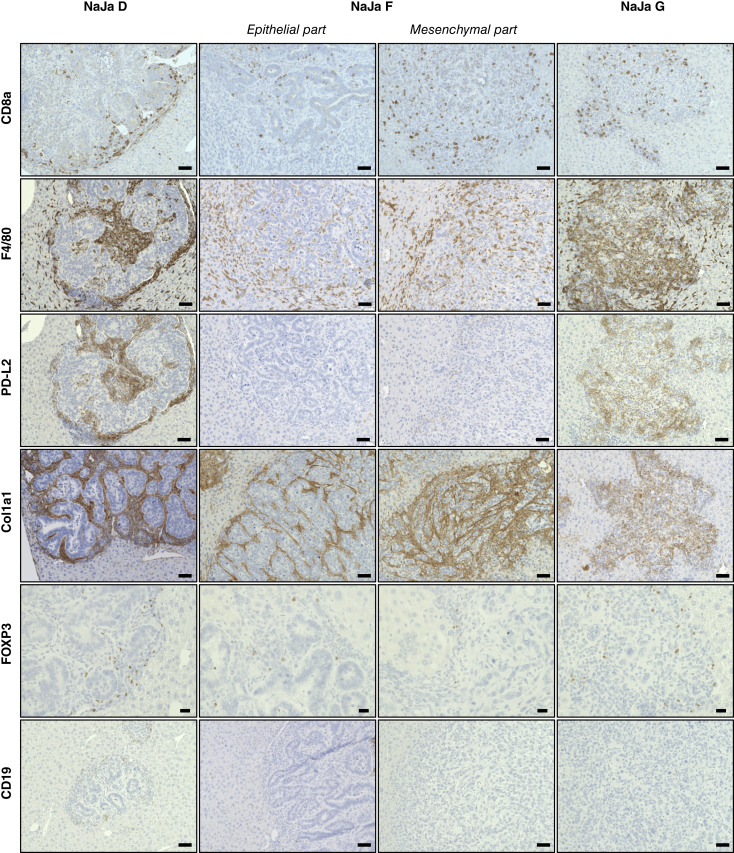
Tumors formed by the three NaJa lines exhibit differences in their TMEs. Representative photos of IHC staining in NaJa-induced liver tumors with indicated antibodies, CD8a, CD8^+^ T cells, F4/80, macrophages, Col1a1, collagen type 1 alpha 1 chain, FOXP3, regulatory T cells, CD19, B cells. Scale bar (FOXP3), 20 μm, scale bar (rest), 50 μm. F4/80 and PD-L2 staining were performed on 3-μm-thick serial cuts to allow comparison of staining patterns in adjacent tumor regions.

Human MSS^+^ BRAF^V600E^–driven colorectal cancer hardly respond to immunotherapies (19), highlighting the need to better understand their TME. A major advantage of the MSS^+^ NaJa model is the possibility to investigate the immune cell infiltrate of BRAF^V600E^-driven colorectal cancer lesions in syngeneic immunocompetent mice. Remarkably, our PVI *in vivo* approach revealed that CD8^+^ T cells—although present in tumors derived from all three cell lines—differed in location and distribution (Fig. 5; Supplementary Fig. S13). In the epithelial crypt-like NaJa-D–derived tumors, CD8^+^ T cells clustered at tumor margins possibly unable to fully infiltrate the tumor tissue. In contrast, CD8^+^ T cells were more evenly distributed in NaJa-G– and NaJa-F–derived tumors. Furthermore, F4/80^+^ macrophages were present in the liver parenchyma but highly abundant in or around tumors. Strikingly, smaller NaJa-D lesions presented with a capsule-like deposit around their invasive margins. This pattern was less pronounced in NaJa-F–derived tumors, in which the macrophages were more loosely dispersed, which might explain their better CD8^+^ T-cell infiltration, as the F4/80^+^ macrophages might provide a physical and immunosuppressive barrier around the tumor. This is further supported by the strong expression of the immune checkpoint PD-L2 around NaJa-D lesions. Interestingly, PD-L2 seems to be expressed by F4/80^+^ TAMs, which prominently feature in that area, again suggesting an immunosuppressive and tumor-supportive function of this macrophage “girdle.” In contrast, the NaJa-G–derived lesions were strongly infiltrated by F4/80-positive macrophages but exhibited a weaker and more scattered PD-L2 staining throughout the tumor. NaJa-F–derived lesions barely showed any PD-L2^+^ cells (Supplementary Fig. S13). Furthermore, there was a strong collagen (Col1a1) accumulation around and within the tumor tissue of NaJa-D– and also NaJa-F–derived lesions, whereas this was less pronounced in NaJa-G–derived tumors. As mechanical hindrances are increasingly recognized as an obstacle for cellular immunotherapies against solid tumors (78), this represents an interesting area for further studies. Moreover, FOXP3^+^ immunosuppressive regulatory T cells seemed to be more abundant at the edges of the NaJa-D–derived lesions, while being less common and more loosely dispersed in NaJa-F– and -G–derived tumors. This might additionally create a rather immunosuppressive TME, especially in NaJa-D–derived tumors. In contrast, CD19^+^ B cells were barely present.

**Figure 6.**
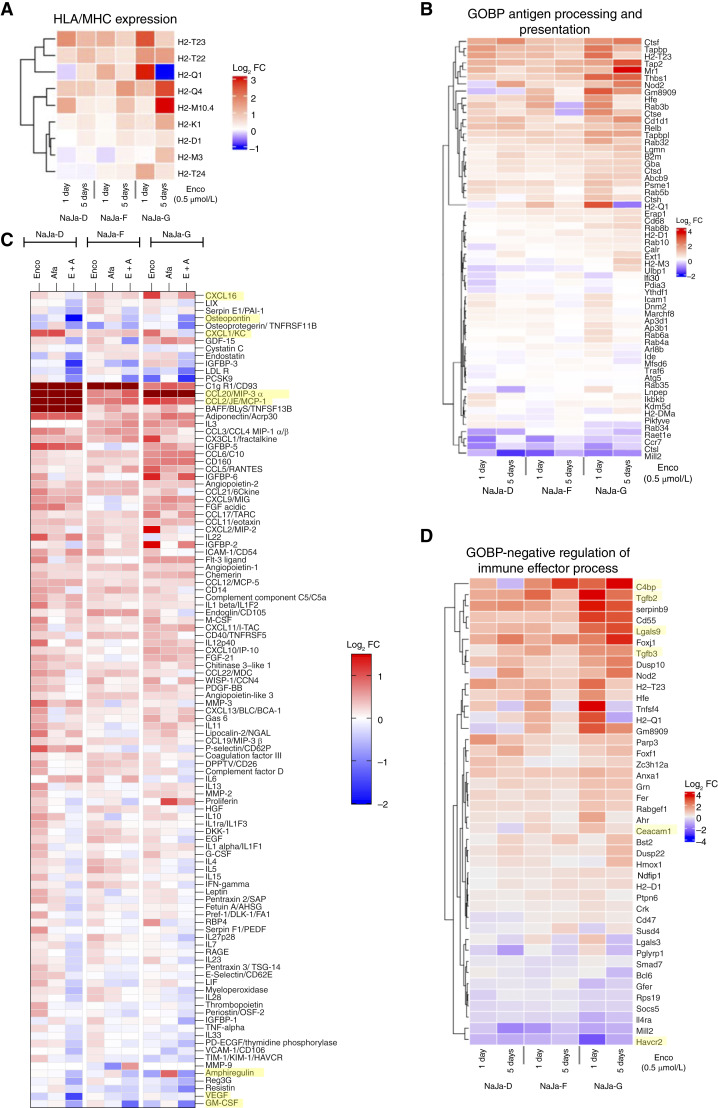
NaJa cells are well suited to study tumor–immune system interactions and their modulation by encorafenib. **A,** Bulk RNA-seq of NaJa cells treated with 0.5 μmol/L encorafenib for either 1 day or 5 days, showing an upregulation of markers for antigen processing and presentation and (**B**) HLA/MHC expression. Shown are the log_2_ FCs of cells treated with encorafenib versus DMSO control. **C,** Cytokine array of NaJa cell supernatant after a 5-day treatment with either 0.5 μmol/L encorafenib, 1 μmol/L afatinib, or a combination of both (E + A). Shown are the log_2_ FCs to a DMSO control. Array membranes and cell line–specific heatmaps are displayed in Supplementary Fig. S15. Discussed cytokines are highlighted in yellow. **D,** Additional heatmap of bulk RNA-seq of encorafenib-treated NaJa cells, showing upregulation or downregulation of transcripts of the GOBP-negative regulation of immune effector process. The genes that are represented have an adjusted *P* value of <0.01 in at least two of the six conditions. Genes of interest are highlighted in yellow.

In addition, solid tumors escape the immune system by impaired antigen processing or presentation as a result of oncogenic signaling events and epigenetic processes (79). Although studies in melanoma demonstrated that ERK pathway inhibitors can be leveraged to induce MHC-mediated antigen presentation (76, 80), very little is known whether these drugs elicit similar effects in colorectal cancer, in particular as this tumor entity is driven by multiple oncogenic signaling pathways. As evident from our RNA-seq analysis, encorafenib induced multiple factors linked to antigen processing and presentation, as well as HLA/MHC expression *in vitro* (Fig. 6A and B). Encorafenib not only upregulated transcripts encoding the classic MHC class Ia molecules presenting peptides to CD8^+^ cytotoxic T cells (H2-K1/H2-D1) but also for lesser understood atypical MHC class Ib proteins (81). This was observed in all three lines but particularly evident in NaJa-G. Encorafenib also induced transcripts for unconventional T-cell receptor ligands like MR1, which is recognized by recently identified MR1 T cells and is emerging as target in tumor immunology (82), and of CD1D1, an NKT cell ligand (Fig. 6B; ref. 83). Stimulated by these findings, we investigated whether encorafenib, either singly or in combination with afatinib, modulates the expression of HLA/MHC molecules in the human colorectal cancer cell line HT29, a frequently used model for BRAF^V600E^-mutant colorectal cancer (8, 9, 25, 37). As shown in Supplementary Fig. S14A–S14C, BRAF but not EGFR inhibition increased the expression of immunoproteasome components, TAP transporters, and HLA/MHC subunits, including β2-microglobulin and the invariant chain (CD74). Thus, encorafenib potentially improves the recognition of human and murine colorectal cancer cells by cytotoxic immune cells via both conventional and atypical MHC molecules and other ligands.

Cytokines, used here in a broad sense to describe cell–cell communication factors such as typical cytokines but also chemokines and growth factor ligands, play a pivotal role in shaping the TME (84). Therefore, we monitored the impact of encorafenib and afatinib, either alone or in combination, on the cytokine secretion profile of the three NaJa lines (Fig. 6C; Supplementary Fig. S15A and S15B). Overall cytokine secretion was increased by encorafenib, both in terms of FCs and the breadth of the spectrum. Commensurate with its weak impact on proliferation (Fig. 3C and D), afatinib had a lesser but still discernable impact. Interestingly, its combination with encorafenib partly reversed the effect of the latter and reduced the secretion in a variety of cytokines in all cell lines (Fig. 6C), potentially owing to the impaired cellular fitness observed in this setting (Fig. 3). There was a variety of secreted cytokines with the potential to shift the immune landscape toward antitumor responses. For example, the proinflammatory chemokines CCL2 or CCL20 were markedly elevated in all cell lines, particularly in NaJa-D. Moreover, CXCL16, which enhances chemotaxis and recruitment of T cells into human colorectal cancers and whose expression is positively correlated with a better prognosis (85), was increased in NaJa-F and -G. Notably, there was increased secretion of tumor-promoting cytokines such as CXCL1/KC or LIF following encorafenib single treatment, albeit their secretion was substantially reduced by the encorafenib/afatinib combination. Additionally, the secretion of angiogenic cytokines, such as VEGF and osteopontin, often enriched in aggressive cancer types, was reduced. Other “desirable” effects of the combination treatment included a reduction in amphiregulin, an EGF-family member associated with cancer progression and liver metastasis formation in colorectal cancer (86, 87). The secretion of GM-CSF, a cytokine involved in macrophage differentiation and recruitment (84), might explain the strong macrophage infiltration that was observed in the *in vivo* NaJa tumor model. Notably, GM-CSF secretion was substantially reduced by encorafenib alone and even more by the encorafenib/afatinib combination. In summary, encorafenib exerts profound effects on the cytokine expression pattern of the three NaJa cell lines, suggesting that this BRAFi renders them more immunogenic *in vivo* and might favor an immunogenic microenvironment.

To further substantiate this concept, we investigated the encorafenib-induced changes in transcripts associated with the negative regulation of immune effector processes (Fig. 6D). Interestingly, the immune checkpoints PD-L2 (*Pdcd1lg2*) and TIM-3 (*Havcr2*) were significantly suppressed in all three NaJa lines by encorafenib (Fig. 6D; Supplementary Table S4). At the same time, transcripts for the TIM-3 ligands *Lgals9* and *Ceacam1* were upregulated by encorafenib, in particular in NaJa-G. This line also exhibited marked upregulation of other immunosuppressive transcripts such as *C4bp*, *Tgfb2*, and *Tgfb3*, with the latter known to play an important role in the immune evasion of MSS^+^ colorectal cancer (88) and being potentially linked to the mesenchymal appearance of NaJa-G cells *in vitro* and *in vivo*. These data, together with IHC analyses presented in Fig. 5, suggest that the three NaJa lines generate distinct TMEs upon BRAFi treatment and therefore reflect the challenging heterogeneity of human colorectal cancer.

## Discussion

In this study, we report the generation, in-depth characterization, and application of three syngeneic mouse cell lines to study various aspects of metastatic BRAF^V600E^-driven colorectal cancer. These cell lines represent a methodologic advance over organoid models in terms of reliable engraftment. As an initial prerequisite, we focused on thoroughly characterizing the BPAC organoids *in vitro*, followed by orthotopic transplantation to assess their tumorigenic and metastatic potential. Although organoid transplantation successfully induced tumor formation in 44% of recipient mice, the take rate of BPAC organoids was lower than in other immunocompetent orthotopic colon organoid transplantation models driven by oncogenic KRAS (and myristoylated AKT1) and biallelic losses of various tumor suppressors (89, 90). Although the underlying reasons remain speculative, potential explanations could include differences in the injection protocols and the contrasting biological properties of BRAF^V600E^ and KRAS^G12D^ oncoproteins (and of the co-mutated alleles and transgenes). Importantly though, we observed metastasis into the liver, prostate, or the peritoneum in 62.5% of mice bearing primary tumors, highlighting their metastatic potential but at the same time underscoring the challenges of predicting the timing and frequency of macrometastatic growth, two critical parameters for drug intervention studies. Although the metastatic potential of the BPAC organoids is evident, our data tie in with a paucity of reliably metastasizing murine colorectal cancer models (91). At least in the context of oncogenic KRAS, several alterations such as *Trp53*, *Apc*, and *Tgfbr2* mutations or the ectopic expression of an artificially myristoylated AKT must coexist to confer metastatic potential to organoids (92). Likewise, induction of BRAF^V600E^ by itself mostly induces nonmetastatic organoids and adenoma *in vivo*, and if metastases are detected, they usually occur with very long latency (20–22, 24–27, 93–95). Even the combination with mutant *Trp53* increases metastatic potential only slightly (25). Moreover, Lannagan and colleagues (95) reported infrequent liver metastasis of BRAF^V600E^ colon organoids with biallelic deletion of *Tgfbr2*, *Rnf43*, *Znrf3*, and *p16 Ink4a* upon implantation into immunocompromised NOD/SCID gamma mice but no macrometastasis to other sites such as the lung or peritoneum. We propose that BPAC organoids owe their metastatic and colonization potential to their increased fitness conferred by the niche independency bestowed by *Apc* deficiency. Indeed, the NaJa cells, which were isolated from the peritoneal cavity of mice orthotopically implanted with BPAC organoids, immediately grew out under very basic culture conditions lacking all the additives typically used for organoid maintenance. We show that all three NaJa lines, despite their unique features discussed below, form aggressive tumors upon PVI-mediated retransplantation into fully immunocompetent C57BL/6N mice within a short time frame. The ability of the BPAC organoid and the derived NaJa cells to establish metastases at very high efficiency and in various organs underscores the metastatic behavior of these cells, whose genetic driver composition matches an extremely aggressive and poor prognostic colorectal cancer subentity found in humans (24). Such aggressive, and in terms of metastatic colonization highly efficient, colorectal cancer cell lines that can be used in syngeneic mice are scarce. However, they represent a critical prerequisite for establishing cohorts for preclinical drug tests for which model systems with comparable and reliable disease onsets are essential. In fact, only two colorectal cancer cell lines, which meet these requirements, have been developed over the last 45 years and are still widely used in tumor immunology, CT26 and MC38 (96). Both chemically induced lines, however, are not suitable to investigate the effects of BRAF^V600E^-selective RAFi-like encorafenib for the following reasons. First, CT26 contains a KRAS^G12D^ mutation (97), which would trigger the aforementioned paradoxical activation of BRAF^V600E^-selective RAFis (3). Second, as CT26 is derived from an undifferentiated colorectal cancer originating in a BALB/c mouse (98), it would be rejected by C57Bl/6 mice, the strain of choice for most immunologic studies. Third, MC38 cells, which are derived from a grade III adenocarcinoma of a C57BL/6 mouse (99), contain an atypical *Braf*^W487C^ variant of unknown significance (100). To evaluate this interesting observation, we characterized the equivalent human BRAF^W450C^ mutant and investigated its signaling and transformation potential (Supplementary Fig. S16A–S16D). Our data, however, revealed neither increased enzymatic activity nor downstream signaling and transformation potential, suggesting that this mutation, despite the high evolutionary conservation of W487/W450, is rather inert and not expected to induce an active conformation in which BRAF is susceptible to type I^1/2^ compounds (74). Consequently, MC38 is unsuitable to study clinically relevant BRAF^V600E^ inhibitors *in vivo*. Moreover, MC38 rather matches an MSI^+^ colorectal cancer (96, 100) and therefore does not reflect the highly treatment-refractory MSS^+^ BRAF^V600E^ subtype. In contrast, the NaJa lines express unmutated mismatch repair genes, indicating that they correspond to the mismatch repair–proficient/MSS^+^ colorectal cancer subtype. Given the general paucity of syngeneic cancer cell lines driven by clinically relevant and/or druggable alterations on one hand and the increasing number of genetically engineered mice on the other hand, the workflow we established in this study for NaJa cells could encourage similarly genetically defined models for other tumor entities.

Despite many advantages, such as the rapid and reliable onset of tumor growth following inoculation, transplantation models using established conventional cancer cell lines are often criticized for their lack of genomic and environmental heterogeneity (96). This particularly applies to colorectal cancer, as this disease is in many aspects heterogeneous, not only from the perspective of genetic driver mutations and the various subentities (101, 102) but also in terms of the differentiation status and drug responsiveness of the individual tumor. For example, two of the most commonly used human MSS^+^ BRAF^V600E^-positive cell lines, HT29 and Colo-205, not only differ drastically in their morphology but also in their responsiveness to BRAF inhibition or depletion (7, 37, 103), with the latter being quite sensitive to and even used to benchmark the efficacy of the first BRAF^V600E^ inhibitors (104, 105). Heterogeneity in initial responses to BRAFis, either singly in the first clinical trials or later in combination with EGFR blockade, was also observed (12, 106). This indicates that BRAF^V600E^-driven colorectal cancers differ in their intrinsic drug resistance and highlights the need of combination therapies informed by the private spectrum of co-mutations and, potentially, epigenetic modifications. Interestingly, the three NaJa cell lines, which originate from the same organoid culture, recapitulate this heterogeneity by displaying various sensitivities to RAFis. This finding is of particular interest as our WES data did not identify obvious genetic alterations explaining this and other phenotypes, such as the contrasting morphologic features displayed by the three NaJa cells *in vitro* and *in vivo*. Thus, NaJa cells might serve as a useful tool to uncover phenotypic plasticity mediated by cell-intrinsic nongenetic events, which are increasingly recognized as contributors to drug resistance (107). An interesting area for future research would be the investigation of whether the contrasting drug sensitivities are functionally linked to the distinct morphologic features. Indeed, our data showing that the mesenchymal NaJa-G cells display a much lower expression of EGFR family members but a higher sensitivity to BRAFis or MEKis in a “monotherapy setting” support a connection between differentiation state and drug responsiveness. This concept is reminiscent to the contrasting difference in BRAFi sensitivity displayed by human colorectal cancer and melanoma lines (8) and would also explain as to why NaJa-G cells, despite their mesenchymal phenotype that is usually associated with drug resistance (108), exhibit high sensitivity to encorafenib, vemurafenib, exarafenib, or trametinib. It will be also of interest to investigate how the distinct NaJa cell phenotypes develop under chronic exposure to targeted therapy compounds. Moreover, the three distinct differentiation states displayed by the NaJa lines *in vitro* seem to influence the heterogeneity of tumor morphology and TME composition *in vivo*. For example, NaJa-D forms CDX2^+^ glandular structures that seem to be encapsulated by prominent collagen I deposition, which might explain the accumulation of PD-L2^+^ macrophages and CD8^+^ T lymphocytes at the tumor/liver parenchyma margin. In contrast, both immune cell types are scattered throughout NaJa-G tumors. Thus, the NaJa cell PVI model might allow to study the heterogeneity of tumor–stroma interactions and the physical and molecular determinants of immune cell infiltration.

Despite their distinct phenotypes, the three NaJa lines share critical features. They all engraft into syngeneic hosts with high efficiency and respond to drugs used to treat human BRAF^V600E^-driven colorectal cancer with similar albeit not identical signaling pathway responses. Examples are the shutdown of immediate early genes, the upregulation of compensatory signaling pathways, and the induction of various processes that are of particular interest from an oncoimmunologic perspective. For example, encorafenib increased transcripts associated with antigen processing and presentation and various proinflammatory cytokines in all three NaJa lines and in human HT29 cells, indicating a conserved immunosuppressive mechanism of BRAF^V600E^. Interestingly, patients with colorectal cancer, who responded to a combination of an anti–PD-1 antibody, dabrafenib, and trametinib for more than 6 months, exhibited increased levels of this transcript category (71). We also showed that the colony formation of all three NaJa lines is strongly compromised by the encorafenib/afatinib combination treatment. As afatinib/BRAFi combinations are active against human colorectal cancer cell lines (7), this pan-EGFR inhibitor should be considered for the treatment of human BRAF^V600E^-driven colorectal cancer, in particular for cases in which no (adequate) response to the BEACON concept is observed. Likewise, the new pan-RAFi exarafenib, despite its slightly higher preference for dimeric class II BRAF mutants (72), showed promising efficacy against all three NaJa lines.

In summary, we developed and extensively characterized three novel syngeneic mouse cell lines to explore various aspects of metastatic BRAF^V600E^-driven colorectal cancer. The NaJa cell lines were engineered with three genetic alterations matching to an aggressive and highly metastatic colorectal cancer subentity found in humans (24). Despite the *Trp53* and *Apc* alterations, however, all three lines respond to clinically used (B)RAFis and MEKis and recapitulate resistance mechanisms against these compounds observed in human colorectal cancer. Therefore, they seem well-suited to explore novel treatment rationales combining these compounds with immune checkpoint therapies in fully immunocompetent hosts. Such experiments will reveal whether ERK pathway inhibitors alter the pattern and spectrum of immune cell infiltrates or improve tumor cell recognition by the immune system, as it is also suggested by first clinical data (71). Addressing this question in this experimentally tractable syngeneic mouse model might inform the rational design of clinical trials combining kinase inhibitors and immunotherapeutics. Given their easy handling and, compared with organoids, cheap propagation, we envisage that they could serve as a platform for further manipulation, such as the editing of genes involved in drug resistance or immune escape.

## Supplementary Material

Supplementary Table S1 List of antibodies used in this study

Supplementary Table S2 Whole exome sequencing data

Supplementary Table S3 Table S3. Apoptosis hallmark genes in RNA-seq of encorafenib-treated NaJa cells

Supplementary Table S4 Table S4. Relative expression of PD-L2 (Pdcdc1lg2) in RNA-seq of encorafenib-treated NaJa cells

Supplementary Data Source data (uncropped blots and tables with individual data points) provided as a single Excel file

Supplementary Figure S1 Supplementary Figure S1 shows that the expression of BRAFV600E and p53R172H as well as Apc deficiency induces distinct morphological changes and transcriptome profiles in murine organoids.

Supplementary Figure S2 Supplementary Figure S2 shows the effect of encorafenib, afatinib and trametinib treatment on BPAC organoid viability.

Supplementary Figure S3 Supplementary Figure S3 shows the take rate of orthotopic transplantation of BPAC organoids, PCR confirmation of tumor origin and H&E and IHC images of tumors and metastases.

Supplementary Figure S4 Supplementary Figure S4 shows the presence of the expected driver mutations in the peritoneal lavages of recipient mice D, F, and G via PCR, as well as allele frequency via WES and a comparison of NaJa cell proliferation.

Supplementary Figure S5 Supplementary Figure S5 shows a Circos plot of the whole-exome sequencing data from NaJa cells and their donor organoid.

Supplementary Figure S6 Supplementary Figure S6 shows the viability of NaJa cells after binimetinib or trametinib treatment as measured by XTT assay.

Supplementary Figure S7 Supplementary Figure S7 shows the membranes from the phospho-RTK array of NaJa cells and detailed cell line specific heatmaps.

Supplementary Figure S8 Supplementary Figure S8 shows comparisons of the baseline transcriptome of NaJa cells, as well as a Western blot of baseline phospho-AKT expression.

Supplementary Figure S9 Supplementary Figure S9 shows the induction of apoptosis hallmark transcripts following encorafenib treatment and a drug washout colony formation assay after encorafenib and afatinib treatment of NaJa cells.

Supplementary Figure S10 Supplementary Figure S10 shows Western blots, colony formation assays, and synergy scores of NaJa cells treated with exarafenib either alone or in combination with afatinib.

Supplementary Figure S11 Supplementary Figure S11 shows images of NaJa cell–induced tumor growth outside the liver.

Supplementary Figure S12 Supplementary Figure S12 shows bioluminescence measurements of the luciferase-transduced NaJa-G cell line (NaJa-G_luc^+^) and NaJa-G_luc^+^-induced tumor growth.

Supplementary Figure S13 Supplementary Figure S13 shows IHC quantifications of the immune microenvironment in NaJa cell-induced liver metastases

Supplementary Figure S14 Supplementary Figure S14 shows upregulation of the MHC presentation machinery in HT29 cells assessed by RNA-seq and Western blot analysis.

Supplementary Figure S15 Supplementary Figure S15 shows membranes from the cytokine array and cell line–specific cytokine heatmaps of NaJa cells.

Supplementary Figure S16 Supplementary Figure S16 shows a focus assay, Western blot and kinase assay characterizing the MC38-associated BRAF W450C mutation.

## Data Availability

RNA-seq data are available under the following Gene Expression Omnibus accession numbers: *GSE296998* (https://www.ncbi.nlm.nih.gov/geo/query/acc.cgi?acc=GSE296998): RNA-seq NaJa cells and *GSE296999* (https://www.ncbi.nlm.nih.gov/geo/query/acc.cgi?acc=GSE296999): RNA-Seq BPAC organoids. WES data are available at BioSample with the ID *PRJNA1258934* and the accession numbers SAMN48637661, SAMN48637662, SAMN48637663, and SAMN48637664. Source data (uncropped blots and tables with individual data points) are provided as an Excel file in Supplementary Data.
