# Supplementary material for: Novel Syngeneic Cell Lines for Studying High-Risk BRAFV600E-Driven Colorectal Cancer In Vivo
Source: Cancer Res Commun. 2026 Feb 16;6(2):320–39. doi: 10.1158/2767-9764.CRC-25-0599 (PMC13037773; doi:10.1158/2767-9764.CRC-25-0599)
Supplement: Supplementary Table S1 — List of antibodies used in this study [file crc-25-0599_supplementary_table_s1_suppst1.docx]

**Supplementary Table S1: Antibodies used in this study**

| **Target** | **Source** | **Identifier** | **RRID** | **Dilution** |
| --- | --- | --- | --- | --- |
| For Western Blot | | | | |
| AKT | Cell Signaling | #9272 | AB_329827 | 1:1000 |
| Phospho-AKT | Cell Signaling | #9271 | AB_329825 | 1:1000 |
| E-Cadherin | BD Bioscience | #610181 | AB_397580 | 1:1000 |
| EGFR | Cell Signaling | #4267 | AB_2895042 | 1:2000 |
| Phospho-EGFR | Cell Signaling | #3777 | AB_2096270 | 1:1000 |
| EPHA2 | Cell Signaling | #6997 | AB_10827743 | 1:1000 |
| Phospho-EPHA2 | Cell Signaling | #12677 | AB_2797989 | 1:2000 |
| ERK | Cell Signaling | #4695 | AB_390779 | 1.1000 |
| Phospho-ERK | Cell Signaling | #4370 | AB_2315112 | 1:1000 |
| HER2 | Cell Signaling | #4290 | AB_10557104 | 1:1000 |
| HER3 | Cell Signaling | #12708 | AB_2721919 | 1:1000 |
| HSP90 | Cell Signaling | #4874 | AB_2121214 | 1:1000 |
| MHC class I | Cell Signaling | #88274 | AB_3714874 | 1:1000 |
| MEK | Cell Signaling | #9122 | AB_823567 | 1:1000 |
| Phospho-MEK | Cell Signaling | #9121 | AB_331648 | 1:2000 |
| N-Cadherin | Cell Signaling | #4061 | AB_10694647 | 1:1000 |
| PSMB8 | Cell Signaling | #13635 | AB_2744693 | 1:1000 |
| TAP1 | Cell Signaling | #49671 | AB_3720472 | 1:1000 |
| TAP2 | Cell Signaling | #25657 | AB_3720473 | 1:1000 |
| Vimentin | BD Bioscience | #550513 | AB_393716 | 1:1000 |
| ZEB1 | Sigma Aldrich | #HPA027524 | AB_1844977 | 1:1000 |
| 14-3-3 | Santa Cruz | sc-1657 | AB_626618 | 1:1000 |
| For Immunohistochemistry and Immunofluorescence | | | | |
| CDX2 | Cell Signaling | #12306 | AB_2797879 | 1:100 |
| CD8a | Invitrogen | 14-0808-82 | AB_2572861 | 1:100 |
| CD19 | Cell Signaling | #90176 | AB_2800152 | 1.1600 |
| Col1a1 | Cell Signaling | #72026 | AB_2904565 | 1:200 |
| DUSP6 | abcam | ab76310 | AB_1523517 | 1:75 |
| E-Cadherin | BD Laboratories | 610181 | AB_397580 | 1:50 |
| F4/80 | Cell Signaling | #70076 | AB_2799771 | 1:125 |
| FOXP3 | Cell Signaling | #12653 | AB_2797979 | 1:400 |
| Ki-67 | Cell Signaling | #9129 | AB_2687446 | 1:400 |
| PD-L2 | Cell Signaling | #49189 | AB_3720474 | 1:100 |
| Alexa Fluor 546 goat anti-mouse IgG | Invitrogen | A11003 | AB_2534071 | 1:200 |
| Alexa Fluor 488 goat anti-rabbit IgG | Invitrogen | A11008 | AB_143165 | 1:200 |
