# Supplementary material for: Novel Syngeneic Cell Lines for Studying High-Risk BRAFV600E-Driven Colorectal Cancer In Vivo
Source: Cancer Res Commun. 2026 Feb 16;6(2):320–39. doi: 10.1158/2767-9764.CRC-25-0599 (PMC13037773; doi:10.1158/2767-9764.CRC-25-0599)
Supplement: Supplementary Figure S1 — shows that the expression of BRAFV600E and p53R172H as well as Apc deficiency induces distinct morphological changes and transcriptome profiles in murine organoids. [file crc-25-0599_supplementary_figure_s1_suppsf1.pdf]

Supplementary Figure S1

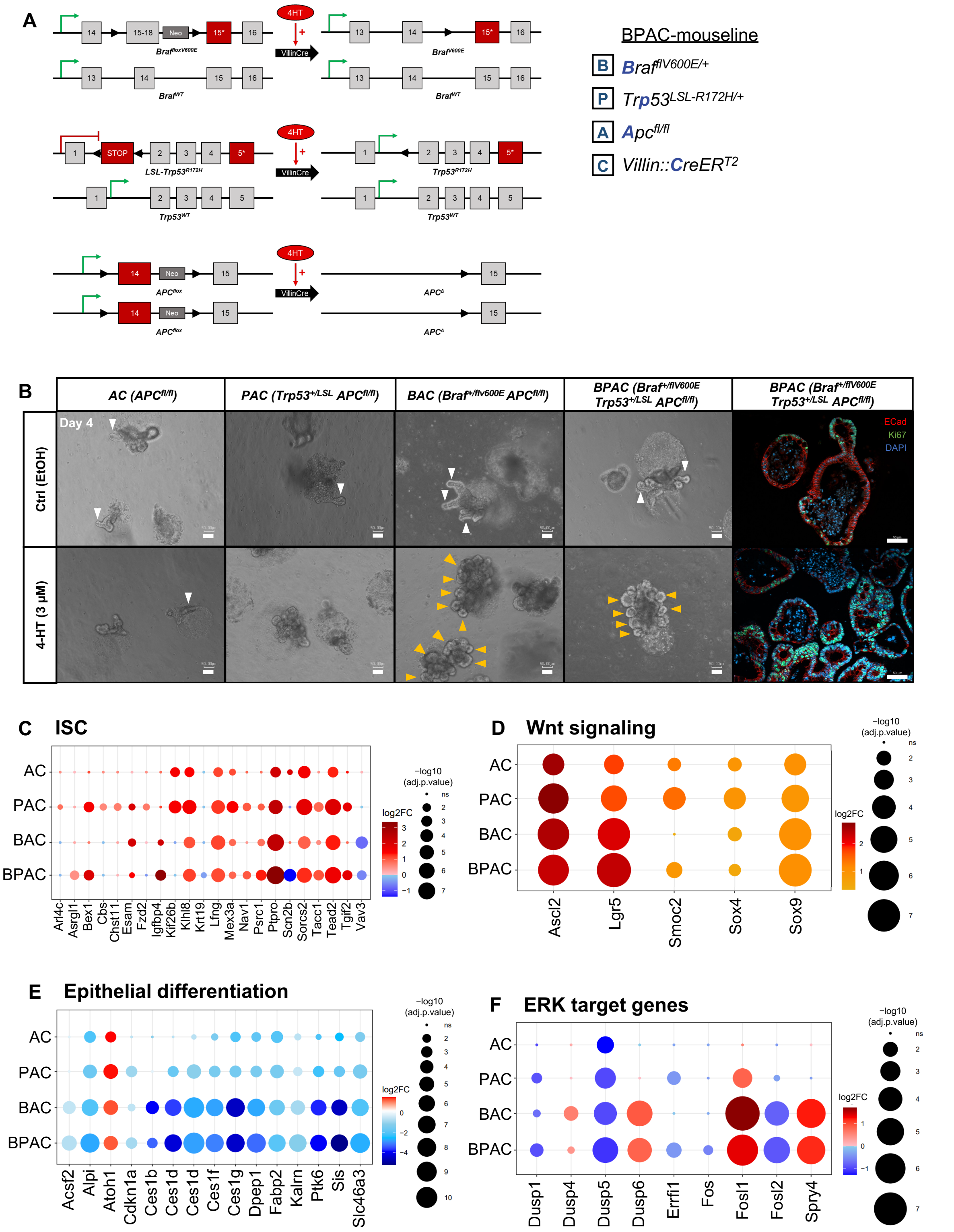

Supplementary Figure S1. Expression of BRAF^{V600E} and p53^{R172H} as well as *Apc* deficiency induce distinct morphological changes and transcriptome profiles. (A) Schematic model of the conditional *Braf^{floxV600E}* (33) and *Trp53$^{LSL-R172H}$* (109) knock-in alleles as well as the *Apc^{flox}* knock-out allele (110). The *Braf^{floxV600E}* allele contains a minigene

corresponding to exon 15 to 18 of wildtype BRAF flanked by two *lox*P sites (black triangles) followed by a mutated exon 15 encoding for the V600E mutation. Following Cre-mediated excision of the minigene, the mutant exon 15 becomes part of the *Braf* open reading frame (ORF), leading to expression of BRAF^{V600E}. The *Trp53*$^{LSL-R172H}$ allele contains a transcriptional STOP cassette flanked by two *lox*P sites (also known as *lox*P-STOP-*lox*P (LSL) cassette) and a mutant exon 5 harboring the R172H mutation. Upon Cre activation, the LSL cassette is removed and an ORF for mutant p53^{R172H} is restored (109). The *Apc*^{flox} knock-out allele carries a *lox*P flanked ("floxed") exon 14, whose Cre mediated deletion disrupts the ORF of *Apc*, resulting in loss of tumor suppressor function (110). **(B)** Control (Ctrl) organoids expressing BRAF^{WT} protein developed longitudinally growing crypts (white arrowheads) with a strongly confined Ki-67 expression. BRAF^{V600E} induced a small and round crypt shape, while the number of crypts increased (yellow arrowheads) and Ki-67 expression was no longer restricted to crypt areas. Brightfield and IF images were taken four days following a 24h pulse with 3 µM 4-HT or EtOH (vehicle control). Scale bars = 50 µm (**C-F**) Bubble plots of bulk RNAseq of 4-HT treated BPAC organoids showing changes in expression of markers of the intestinal stem cell (ISC) niche (C), Wnt signaling (D), epithelial differentiation (E) and ERK target genes (F). Each bubble plot displays the log2-fold change and significance (adjusted p-value) of the indicated transcripts in 4-HT treated organoids vs. EtOH treated organoids. For each genotype, organoid clones derived from two donor mice were used from two distinct passages. Shown is the mean of transcript abundance from these four data points.
