# Supplementary material for: Novel Syngeneic Cell Lines for Studying High-Risk BRAFV600E-Driven Colorectal Cancer In Vivo
Source: Cancer Res Commun. 2026 Feb 16;6(2):320–39. doi: 10.1158/2767-9764.CRC-25-0599 (PMC13037773; doi:10.1158/2767-9764.CRC-25-0599)
Supplement: Supplementary Figure S2 — shows the effect of encorafenib, afatinib and trametinib treatment on BPAC organoid viability. [file crc-25-0599_supplementary_figure_s2_suppsf2.pdf]

Supplementary Figure S2

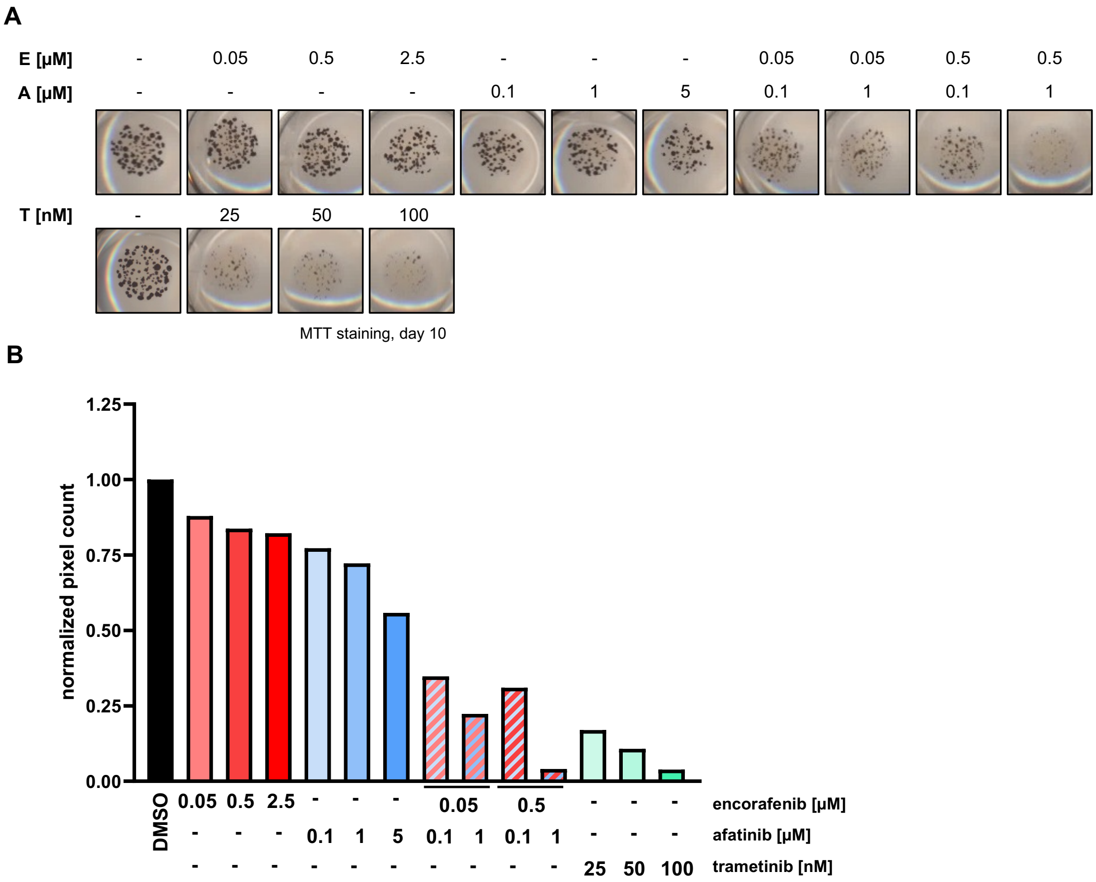

Supplementary Figure S2. **Combined inhibition of BRAF^{V600E} and HER family receptors reduces viability in BPAC organoids.** (**A**) Representative MTT staining of 4-HT induced BPAC organoids treated with DMSO as a vehicle control or the indicated inhibitors (E = encorafenib, A = afatinib, T = trametinib) over the course of ten days. Dark blue colonies indicate metabolic activity. (**B**) Quantification of the data shown in (A). Pixel count was normalized to corresponding DMSO control. Data represent the mean of three technical replicates.
