# Supplementary material for: Novel Syngeneic Cell Lines for Studying High-Risk BRAFV600E-Driven Colorectal Cancer In Vivo
Source: Cancer Res Commun. 2026 Feb 16;6(2):320–39. doi: 10.1158/2767-9764.CRC-25-0599 (PMC13037773; doi:10.1158/2767-9764.CRC-25-0599)
Supplement: Supplementary Figure S3 — shows the take rate of orthotopic transplantation of BPAC organoids, PCR confirmation of tumor origin and H&E and IHC images of tumors and metastases. [file crc-25-0599_supplementary_figure_s3_suppsf3.pdf]

Supplementary Figure S3

A

mouse		primary tumor (colon)	metastasis	days
**1**		✓	liver	63
**2**		✗	✗	-
**3**		✗	✗	-
**4**		✓	prostate	103
**5**	*A*	✓	✗	103
**6**	*B*	✓	✗	103
**7**	*C*	✓	✗	147
**8**	*D*	✓	peritoneum	147
**9**	*E*	✗	✗	147
**10**	*F*	✓	peritoneum	147
**11**	*G*	✓	peritoneum	147
**12**		✗	✗	-
**13**		✗	✗	-
**14**		✗	✗	-
**15**		✗	✗	-
**16**		✗	✗	-
**17**		✗	✗	-
**18**		✗	✗	-

B

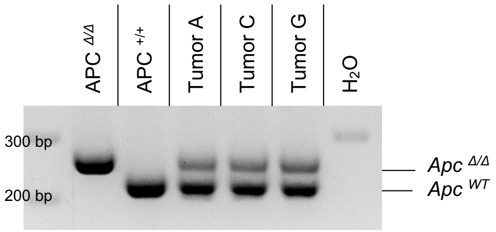

C

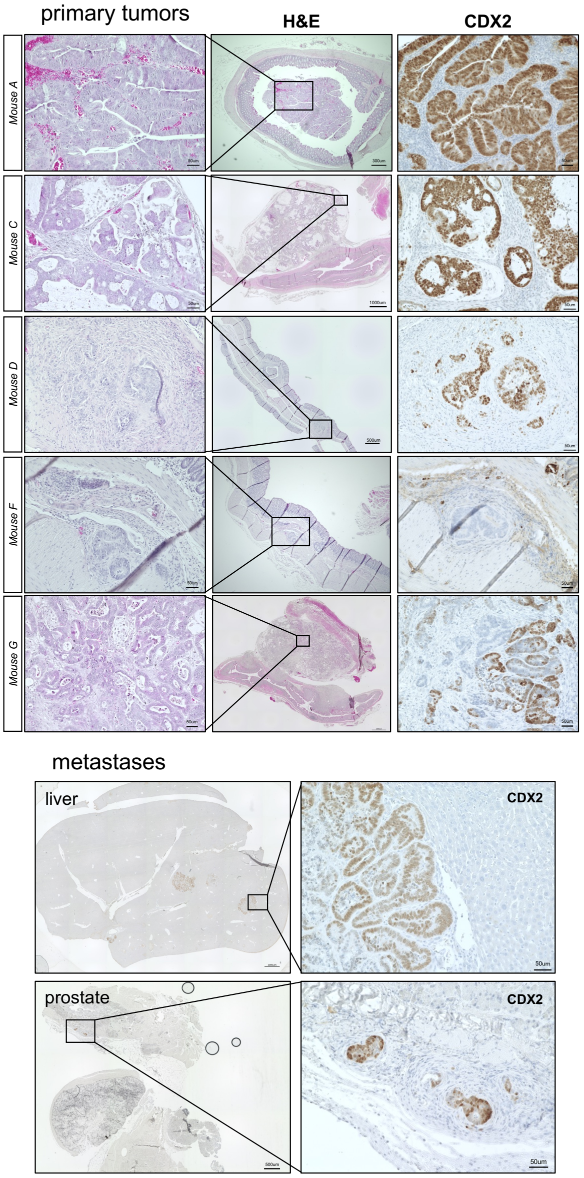

Supplementary Figure S3. **Orthotopic transplantation of BPAC organoids leads to tumor growth and metastases in immunocompetent syngeneic recipient mice.** (**A**) Table summarizing all mice in this study that received recombined BPAC organoids via orthotopic transplantation. Mice with colonoscopy confirmed primary tumors were subject to detailed necropsy, incl. peritoneal lavage and H&E staining of the suspect colon tissue. (**B**) PCRs confirming the presence of the recombined *Apc*$^{\Delta}$, allele in genomic DNA derived from depicted tumor samples. Genomic WT DNA and recombined genomic DNA was used as controls. (**C**) Top: H&E staining and IHC of the intestinal differentiation marker CDX2 (brown staining) of primary tumors derived by orthotopic transplantation of 4-HT induced BPAC organoids. Bottom: liver and prostate metastases, stained for CDX2 of two recipient mice. Scale bar = 50 µm.
