# Supplementary material for: Novel Syngeneic Cell Lines for Studying High-Risk BRAFV600E-Driven Colorectal Cancer In Vivo
Source: Cancer Res Commun. 2026 Feb 16;6(2):320–39. doi: 10.1158/2767-9764.CRC-25-0599 (PMC13037773; doi:10.1158/2767-9764.CRC-25-0599)
Supplement: Supplementary Figure S4 — shows the presence of the expected driver mutations in the peritoneal lavages of recipient mice D, F, and G via PCR, as well as allele frequency via WES and a comparison of NaJa cell proliferation. [file crc-25-0599_supplementary_figure_s4_suppsf4.pdf]

Supplementary Figure S4

A

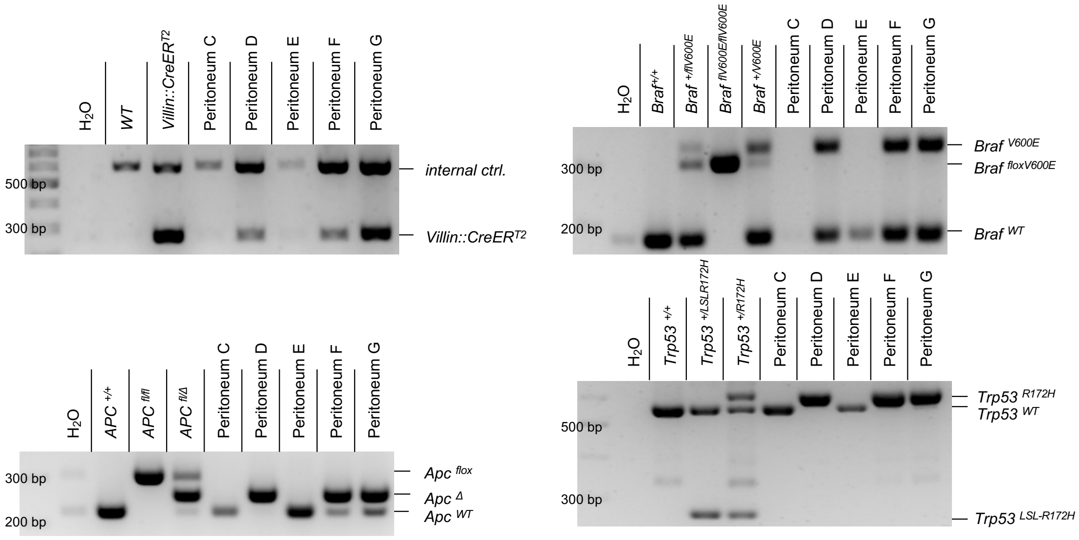

B

Gene	mutation	AF Col235	AF NaJa-D	AF NaJa-F	AF NaJa-G
Trp53	R172H	0.568	0.993	0.994	0.970
Braf	V600E	0.410	0.488	0.455	0.485

AF = allele frequency

C

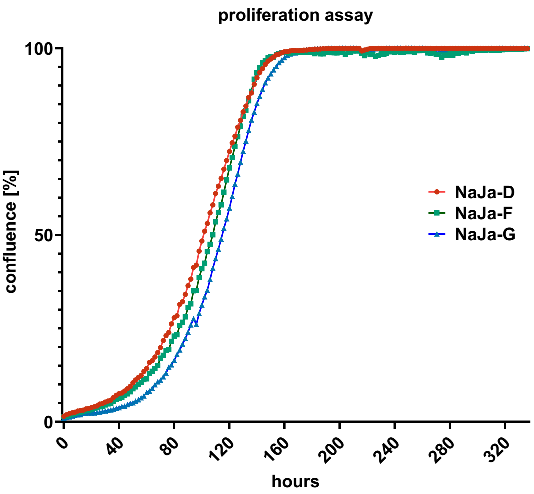

Supplementary Figure S4. Cells outgrowing from peritoneal lavages of three tumor bearing mice carry the expected driver mutations. (**A**) PCRs confirming the presence of the recombined *Apc*$^{\Delta}$, *Braf*^{V600E} and *Trp53*^{R172H} alleles as well as the presence of the *Villin::*CreER^{T2} transgene in peritoneal lavages of recipient mice D, F and G. As controls genomic WT DNA, genomic DNA containing the *lox*P sites (fl/fl) and genomic DNA from recombined BPAC organoids was used. (**B**) Extract of whole exome sequencing (WES), comparing the allele frequency (AF) of the *Trp53*^{R172H} and the *Braf*^{V600E} mutation in donor organoid (Col235) and NaJa cells. (**C**) Proliferation rates of NaJa-D (red), NaJa-F (green) and NaJa-G (blue). The confluence was defined by taking a photo every two hours with the Incucyte® Live-Cell Analysis System over a time period of 14 days. Shown is the mean of four technical replicates for each condition.
