# Supplementary material for: Novel Syngeneic Cell Lines for Studying High-Risk BRAFV600E-Driven Colorectal Cancer In Vivo
Source: Cancer Res Commun. 2026 Feb 16;6(2):320–39. doi: 10.1158/2767-9764.CRC-25-0599 (PMC13037773; doi:10.1158/2767-9764.CRC-25-0599)
Supplement: Supplementary Figure S5 — shows a Circos plot of the whole-exome sequencing data from NaJa cells and their donor organoid. [file crc-25-0599_supplementary_figure_s5_suppsf5.pdf]

Supplementary Figure S5

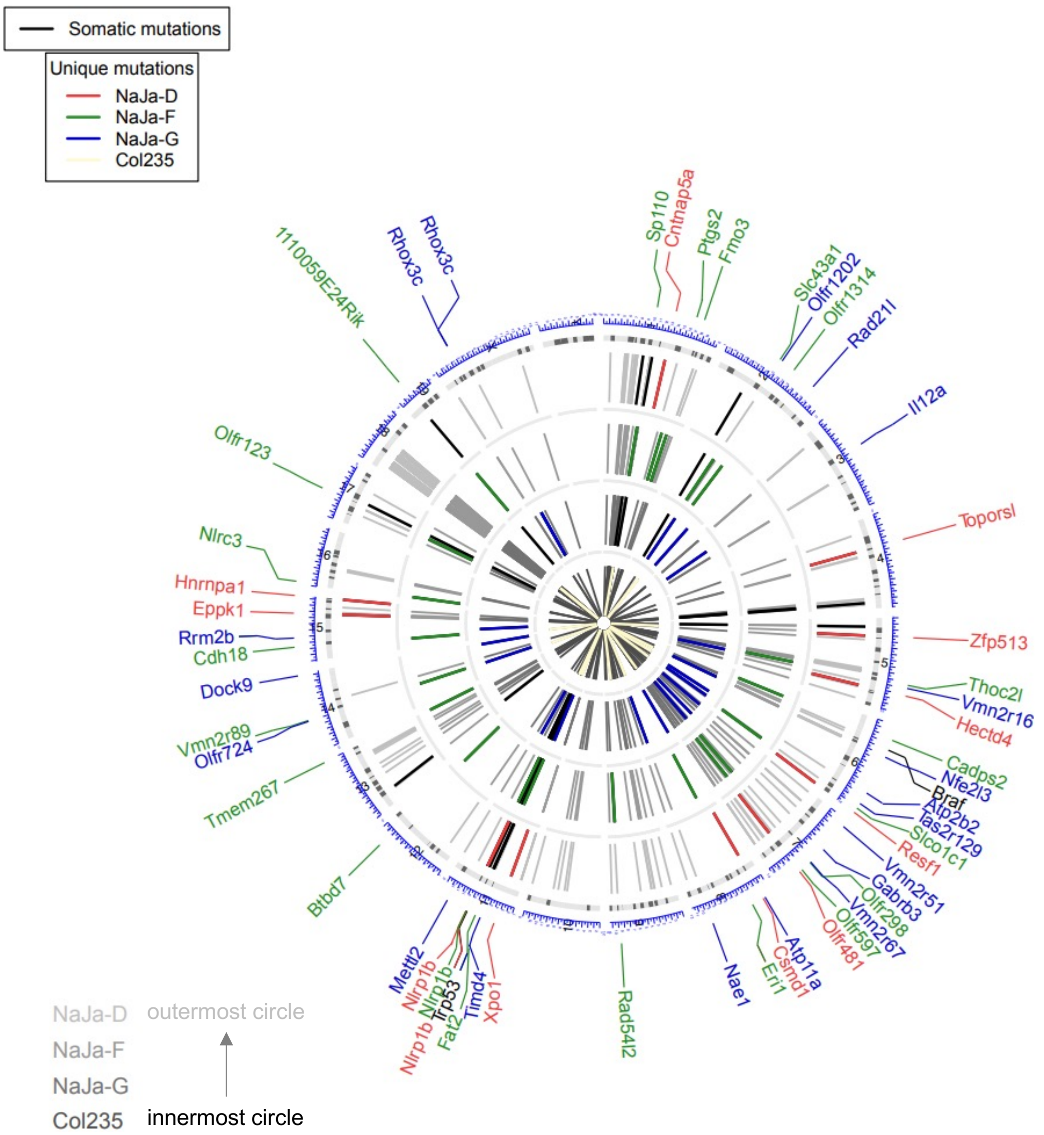

Supplementary Figure S5. Whole exome sequencing of NaJa cells and their donor organoid. (**A**) Circos plot depicting mutations uncovered by WES along the chromosomes per individual NaJa cell line and the donor organoid Col235 (innermost to outermost circle: Col235 > NaJa-G > NaJa-F > NaJa-D). Somatic mutations are marked in black, while unique mutations specific to each sample are indicated by distinct colors (red: NaJa-D, green: NaJa-F, blue: NaJa-G, yellow: Col235).
