# Supplementary material for: Novel Syngeneic Cell Lines for Studying High-Risk BRAFV600E-Driven Colorectal Cancer In Vivo
Source: Cancer Res Commun. 2026 Feb 16;6(2):320–39. doi: 10.1158/2767-9764.CRC-25-0599 (PMC13037773; doi:10.1158/2767-9764.CRC-25-0599)
Supplement: Supplementary Figure S6 — shows the viability of NaJa cells after binimetinib or trametinib treatment as measured by XTT assay. [file crc-25-0599_supplementary_figure_s6_suppsf6.pdf]

Supplementary Figure S6

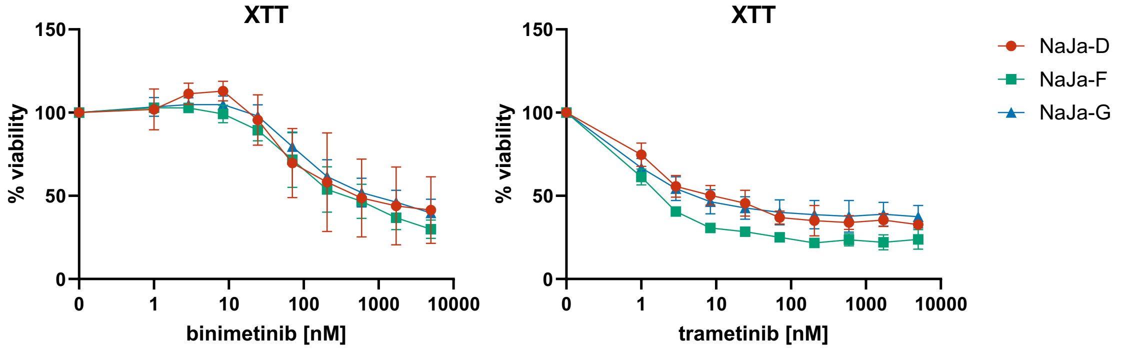

Supplementary Figure S6. Comparison of MEK inhibitor efficiency. NaJa-cells were treated with various concentrations of binimetinib or trametinib over the course of 72 h before viability was measured via XTT staining. Data are presented as mean ± SD. n = 4 for binimetinib, n = 3 for trametinib
