# Supplementary material for: Novel Syngeneic Cell Lines for Studying High-Risk BRAFV600E-Driven Colorectal Cancer In Vivo
Source: Cancer Res Commun. 2026 Feb 16;6(2):320–39. doi: 10.1158/2767-9764.CRC-25-0599 (PMC13037773; doi:10.1158/2767-9764.CRC-25-0599)
Supplement: Supplementary Figure S7 — shows the membranes from the phospho-RTK array of NaJa cells and detailed cell line specific heatmaps. [file crc-25-0599_supplementary_figure_s7_suppsf7.pdf]

Supplementary Figure S7

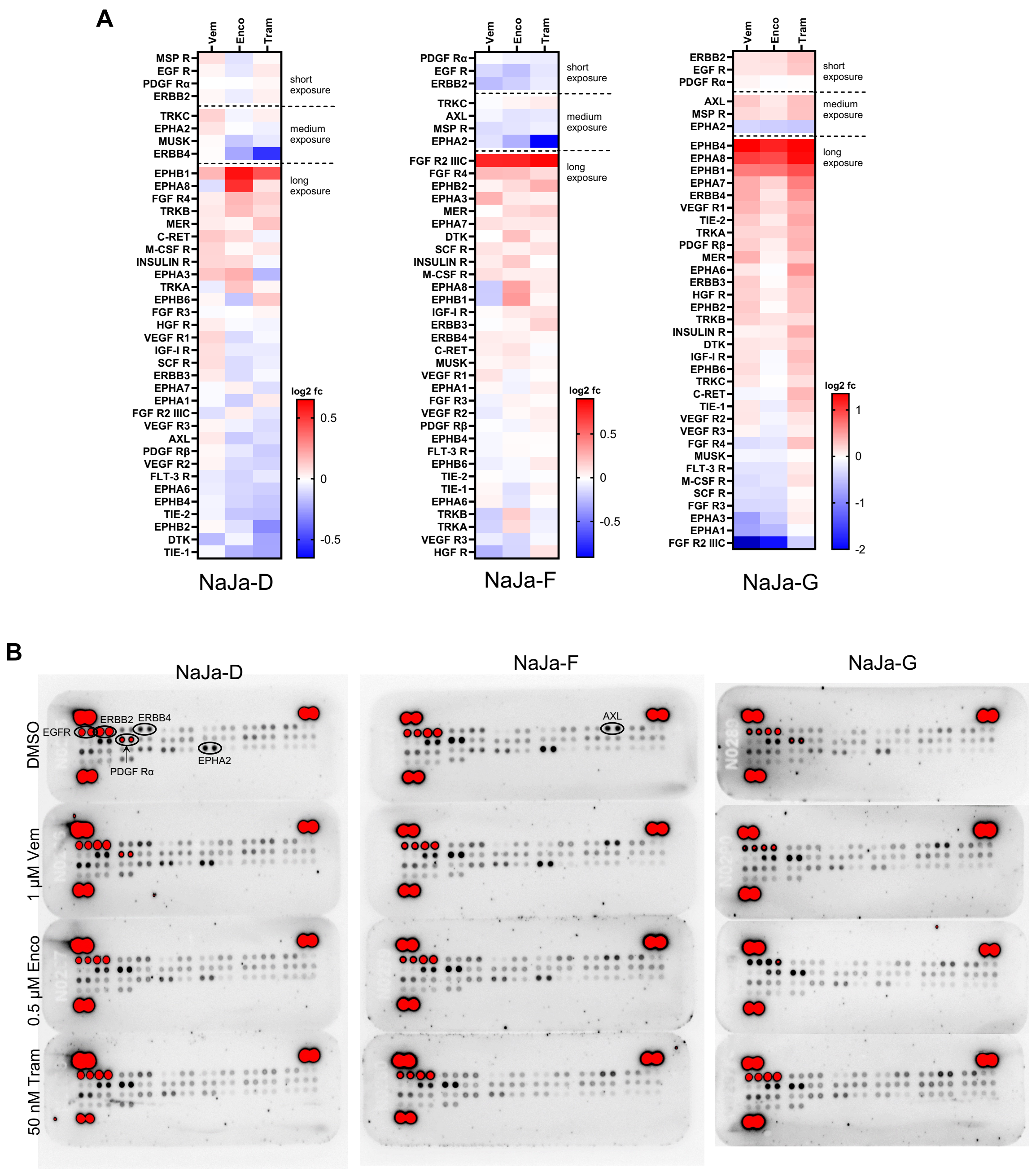

Supplementary Figure S7. **Phospho-RTK array of NaJa cells.** (**A**) Heatmaps of phospho-RTK array of NaJa cells treated with 1 µM vemurafenib (Vem), 0.5 µM encorafenib (Enco) or 50 nM trametinib (Tram) for five days. The heatmaps were clustered singly for each cell line. Shown is the log2-fold change to a DMSO control. The membranes used for the phospho-RTK array (at the longest exposure time) are shown in (**B**). The reference spots indicate that the membrane was incubated with HRP-coupled phospho-tyrosine antibodies.
