# Supplementary material for: Novel Syngeneic Cell Lines for Studying High-Risk BRAFV600E-Driven Colorectal Cancer In Vivo
Source: Cancer Res Commun. 2026 Feb 16;6(2):320–39. doi: 10.1158/2767-9764.CRC-25-0599 (PMC13037773; doi:10.1158/2767-9764.CRC-25-0599)
Supplement: Supplementary Figure S8 — shows comparisons of the baseline transcriptome of NaJa cells, as well as a Western blot of baseline phospho-AKT expression. [file crc-25-0599_supplementary_figure_s8_suppsf8.pdf]

Supplementary Figure S8

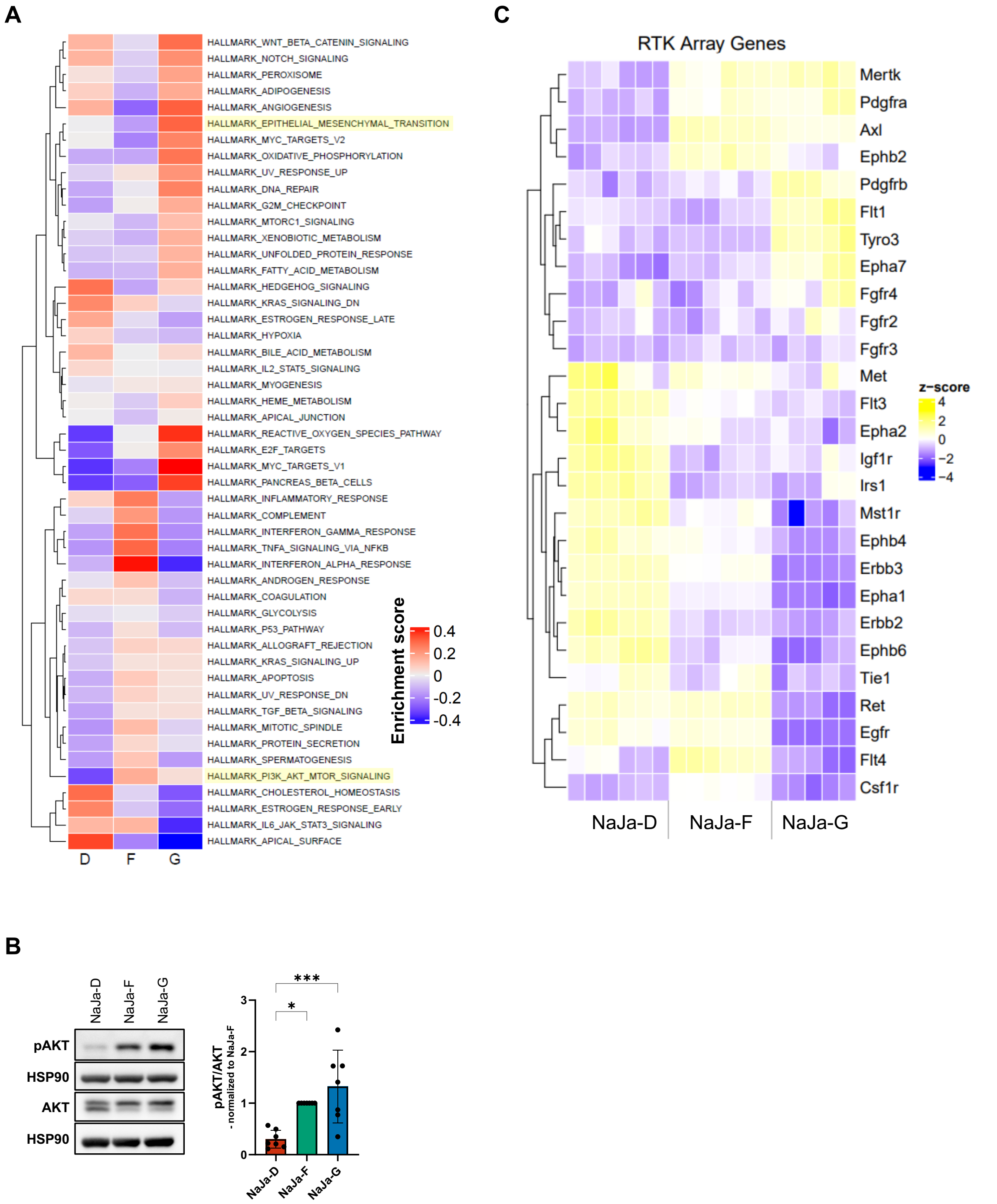

Supplementary Figure S8. Comparison of baseline transcriptome. (**A**) Bulk RNA-seq was conducted with DMSO-treated NaJa cells. Heatmap of NaJa cells (-D, -F, -G) showing the Enrichment score, which reflects the relative activity of Hallmark pathways. Discussed hallmarks are highlighted in yellow. (**B**) WB analysis and quantification of pAKT and total AKT in NaJa cell DMSO controls (n = 7). HSP90 serves as a loading control. Data are presented as mean ± SD and were normalized to NaJa-F for comparison. Statistical significance was calculated using one-way ANOVA and Tukey's multiple-comparisons test. *P <0.05, **P <

0.01, ****P < 0.0001. (**C**) Bulk RNA-seq was conducted with DMSO-treated NaJa cells. Heatmap of z-score for RTK array genes for the NaJa cells (-D, -F, -G) including replicates (n = 6).
