# Supplementary material for: Novel Syngeneic Cell Lines for Studying High-Risk BRAFV600E-Driven Colorectal Cancer In Vivo
Source: Cancer Res Commun. 2026 Feb 16;6(2):320–39. doi: 10.1158/2767-9764.CRC-25-0599 (PMC13037773; doi:10.1158/2767-9764.CRC-25-0599)
Supplement: Supplementary Figure S9 — shows the induction of apoptosis hallmark transcripts following encorafenib treatment and a drug washout colony formation assay after encorafenib and afatinib treatment of NaJa cells. [file crc-25-0599_supplementary_figure_s9_suppsf9.pdf]

Supplementary Figure S9

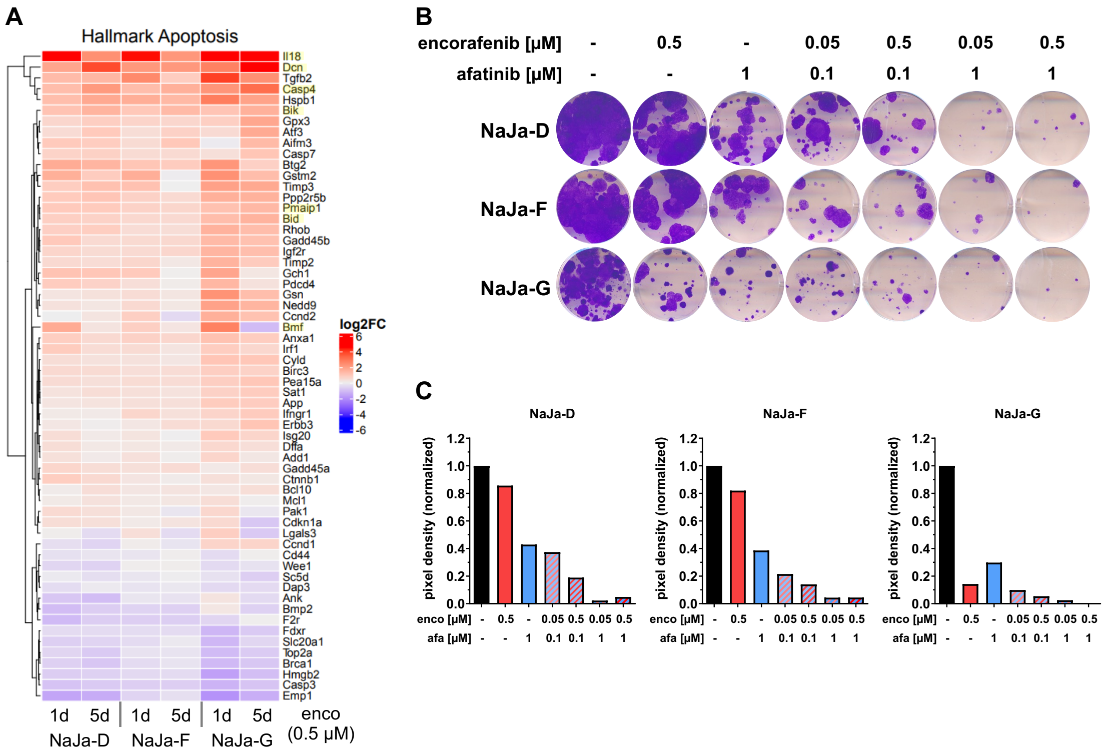

Supplementary Figure S9. Encorafenib induces apoptosis hallmark transcripts, while durable growth suppression requires combination with afatinib. (**A**) Heatmap of bulk RNA-seq of NaJa cells treated with 0.5 µM encorafenib for either one or five days (1d, 5d). Shown are the log2-fold changes of encorafenib treated cells vs. DMSO control of the apoptosis hallmark gene set. The genes that are displayed have an adj. p-value <0.01 in at least 4 of the 6 conditions. Genes of interest are highlighted in yellow. (**B**) Representative images of NaJa-cells, treated with the indicated inhibitor concentrations over the course of ten days before the drugs were washed out and replaced with regular medium. Crystal violet staining was performed five days after wash out to show colony formation. Quantification of the relative pixel density is shown in (**C**). The data shows the mean of three technical replicates, normalized to the DMSO control of each cell line.
