# Supplementary material for: Novel Syngeneic Cell Lines for Studying High-Risk BRAFV600E-Driven Colorectal Cancer In Vivo
Source: Cancer Res Commun. 2026 Feb 16;6(2):320–39. doi: 10.1158/2767-9764.CRC-25-0599 (PMC13037773; doi:10.1158/2767-9764.CRC-25-0599)
Supplement: Supplementary Figure S10 — shows Western blots, colony formation assays, and synergy scores of NaJa cells treated with exarafenib either alone or in combination with afatinib. [file crc-25-0599_supplementary_figure_s10_suppsf10.pdf]

Supplementary Figure S10

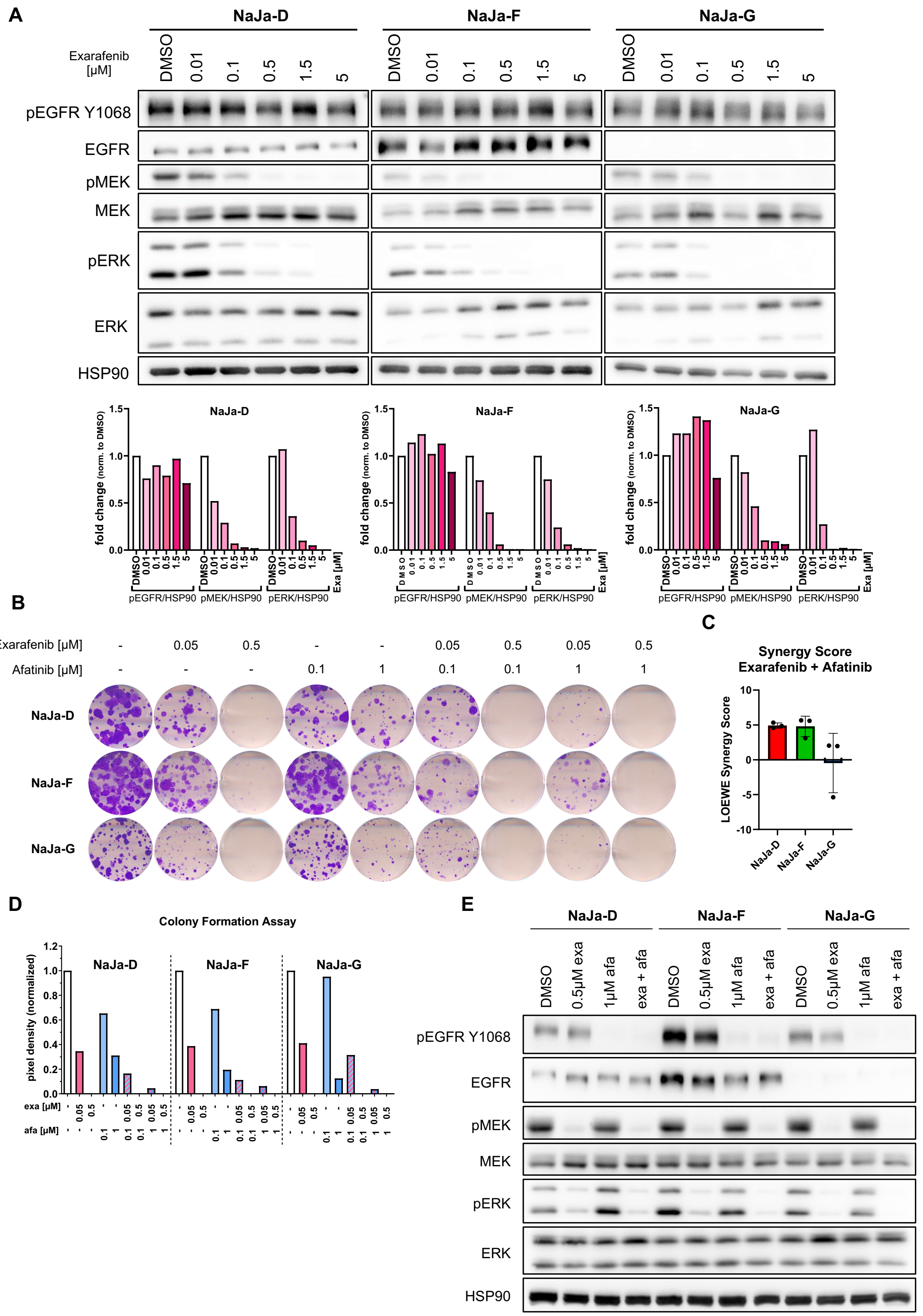

Supplementary Figure S10. Exarafenib induces marked inhibition of ERK signaling and suppresses colony formation in NaJa cells. (**A**) The NaJa cells were treated either with

DMSO or increasing concentrations of the pan-Raf inhibitor exarafenib for 6h before lysis. Western blot was conducted with indicated antibodies. HSP90 serves as a loading control. Densitometric detection of pEGFR, pMEK and pERK for all three cell lines is shown underneath. Protein expression was normalized to corresponding HSP90 loading control and DMSO treated cells. (**B**) Representative images of colony formation assay at day ten. NaJa cells were treated with DMSO or the indicated inhibitor concentrations. (**C**) Quantification of synergy scores of exarafenib and afatinib, as calculated by LOEWE (n = 3). A synergy score between +10 and -10 indicates an additive effect between two drugs. Data are presented as mean ± SD. (**D**) Quantification of the colony formation assays shown in (B). Data are derived from three technical replicates and normalized to DMSO control. (**E**) Western blot of NaJa cells treated either with DMSO or 0.5 µM exarafenib (exa), 1 µM afatinib (afa) or the combination of both (exa + afa) for 24h before lysis. HSP90 serves as a loading control.
