# Supplementary material for: Novel Syngeneic Cell Lines for Studying High-Risk BRAFV600E-Driven Colorectal Cancer In Vivo
Source: Cancer Res Commun. 2026 Feb 16;6(2):320–39. doi: 10.1158/2767-9764.CRC-25-0599 (PMC13037773; doi:10.1158/2767-9764.CRC-25-0599)
Supplement: Supplementary Figure S11 — shows images of NaJa cell–induced tumor growth outside the liver. [file crc-25-0599_supplementary_figure_s11_suppsf11.pdf]

Supplementary Figure S11

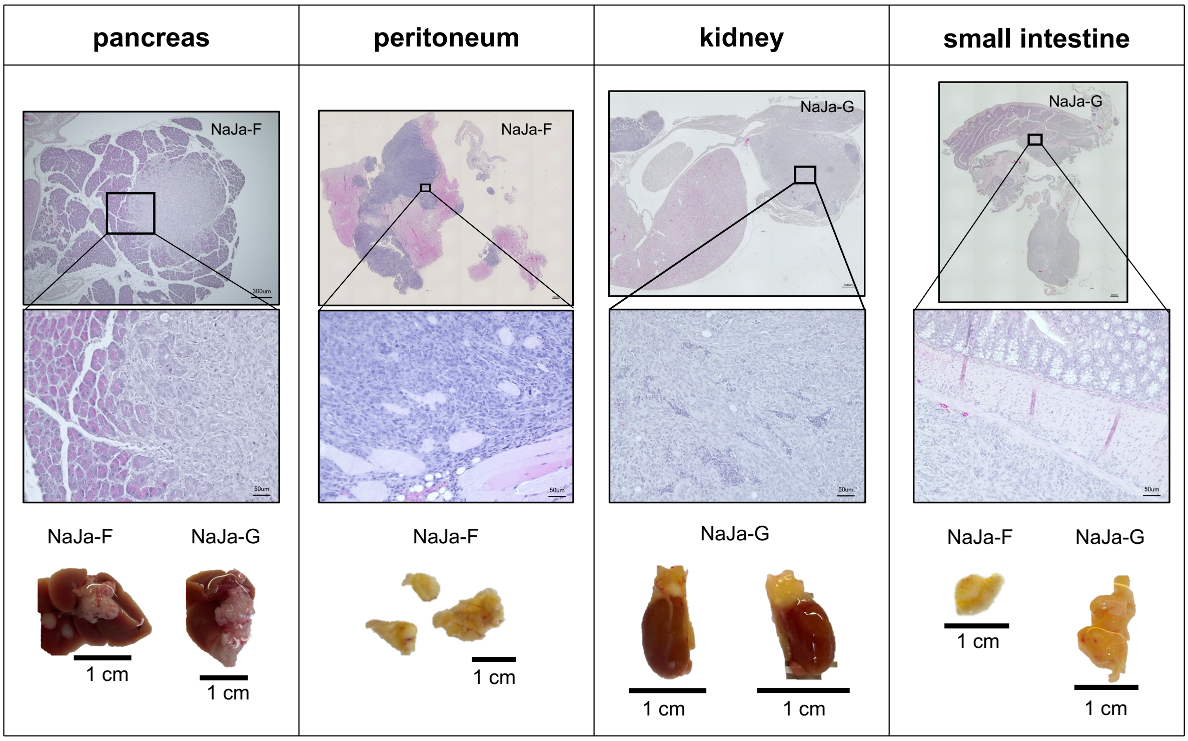

Supplementary Figure S11. NaJa cells induced additional tumor growth outside of the liver. Metastases growth could be detected four weeks post portal vein injection of NaJa cells. Depicted are metastases grown in pancreas, peritoneum and kidney as well as a metastasis colonizing the outer layer of the small intestine. Shown are H&E staining photos of representative sections (scale bar, magnification = 50 µm), as well as photographs of the tumor tissue.
