# Supplementary material for: Novel Syngeneic Cell Lines for Studying High-Risk BRAFV600E-Driven Colorectal Cancer In Vivo
Source: Cancer Res Commun. 2026 Feb 16;6(2):320–39. doi: 10.1158/2767-9764.CRC-25-0599 (PMC13037773; doi:10.1158/2767-9764.CRC-25-0599)
Supplement: Supplementary Figure S12 — shows bioluminescence measurements of the luciferase-transduced NaJa-G cell line (NaJa-G_luc+) and NaJa-G_luc+-induced tumor growth. [file crc-25-0599_supplementary_figure_s12_suppsf12.pdf]

Supplementary Figure S12

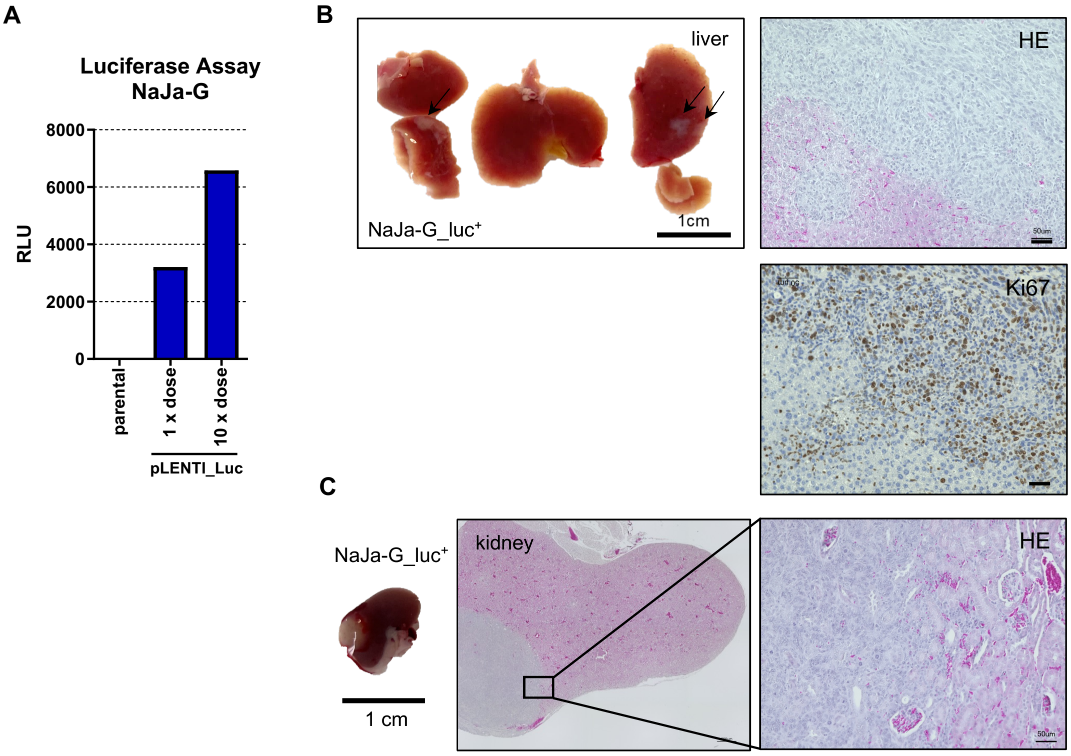

Supplementary Figure S12. Luciferase-transfected NaJa-G cells show luminescence and lead to tumor and metastasis growth. NaJa-G cells were transduced with the *pLENTI_SV40_Lucif_ZEO* construct. (**A**) Measurement of *in vitro* luciferase activity in parental NaJa-G cells vs the transduced NaJa-G (pLENTI_Luc). RLU = relative luminescence units (**B**) The liver tissue of recipient mice, four weeks post injection with pLENTI-Luc transduced NaJa-G cells (NaJa-G_luc$^+$) via the portal vein. Black arrows point toward visible tumor lesions. H&E staining and IHC of the proliferation marker Ki-67 of FFPE sections, showing the NaJa-G_luc$^+$-derived tumors. (**C**) Photo and HE staining of a NaJa-G_luc$^+$-derived kidney metastasis. Scale bar, magnification = 50 µm.
