# Supplementary material for: Novel Syngeneic Cell Lines for Studying High-Risk BRAFV600E-Driven Colorectal Cancer In Vivo
Source: Cancer Res Commun. 2026 Feb 16;6(2):320–39. doi: 10.1158/2767-9764.CRC-25-0599 (PMC13037773; doi:10.1158/2767-9764.CRC-25-0599)
Supplement: Supplementary Figure S13 — shows IHC quantifications of the immune microenvironment in NaJa cell-induced liver metastases [file crc-25-0599_supplementary_figure_s13_suppsf13.pdf]

Supplementary Figure S13

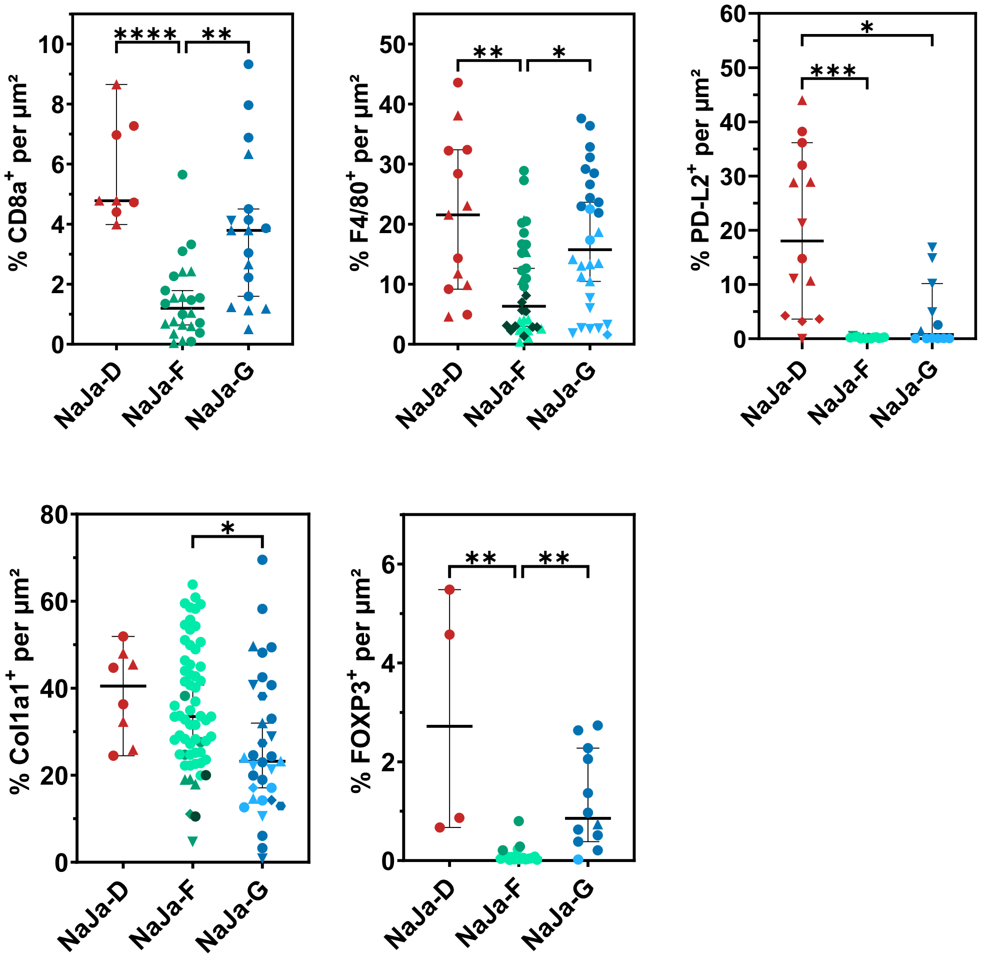

Supplementary Figure S13. IHC Quantifications of the immune microenvironment in NaJa cell-induced liver metastases. IHC staining was performed for CD8a ($CD8^{+}$ T cells), F4/80 (macrophages), PD-L2 (programmed cell death 1 ligand 2), Col1a1 (collagen type I alpha 1 chain), and FOXP3 (regulatory T cells). Each dot represents an individual tumor; different shapes indicate individual tissue sections, and colors denote individual mice. Data are shown as median ± 95% CI. Statistical significance was assessed using the Kruskal–Wallis test followed by Dunn's multiple-comparison test. *P <0.05, **P < 0.01, ***P < 0.001, ****P < 0.0001.
