# Supplementary material for: Novel Syngeneic Cell Lines for Studying High-Risk BRAFV600E-Driven Colorectal Cancer In Vivo
Source: Cancer Res Commun. 2026 Feb 16;6(2):320–39. doi: 10.1158/2767-9764.CRC-25-0599 (PMC13037773; doi:10.1158/2767-9764.CRC-25-0599)
Supplement: Supplementary Figure S14 — shows upregulation of the MHC presentation machinery in HT29 cells assessed by RNA-seq and Western blot analysis. [file crc-25-0599_supplementary_figure_s14_suppsf14.pdf]

Supplementary Figure S14

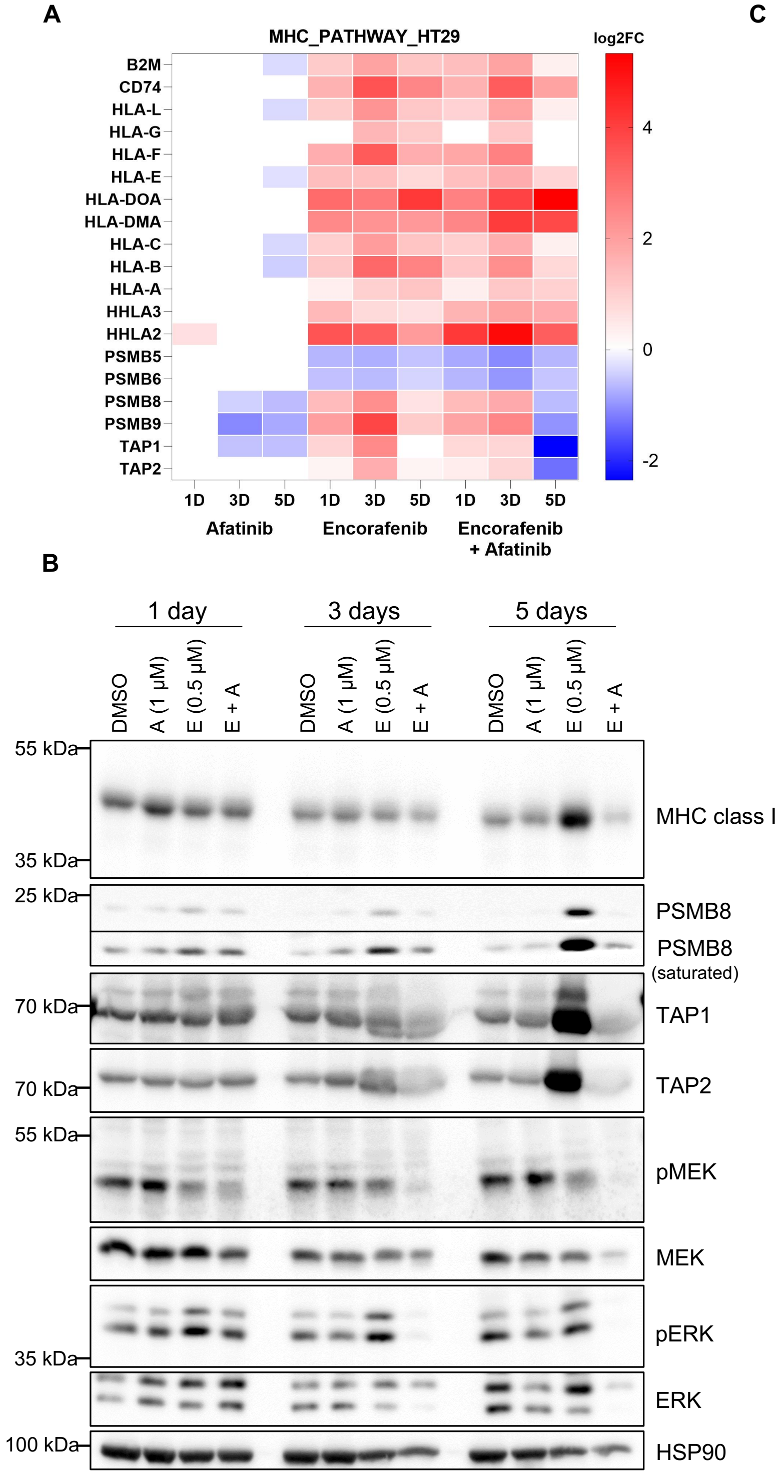

Supplementary Figure S14. Human HT29 cells upregulate MHC presentation after encorafenib treatment. (**A**) Heat map of RNAseq data showing the upregulation of molecules involved in antigen presentation and processing in human HT29 cells following treatment with 0.5 µM encorafenib, either singly or in combination with 1 µM afatinib for one day (1D), three

days (3D) and five days (5D). Note that afatinib alone does not induce significant changes in MHC pathway gene expression. (**B**) Representative Western blot analysis of HT29 cells confirming the upregulation of MHC class I molecules, the proteasome subunit beta type-8 (PSMB8) and Transporter associated with antigen processing 1 (TAP1) and TAP2. Cells were treated as described above. (**C**) Quantification of the data shown in (B). Pixel count was normalized to corresponding DMSO control. Data represent the mean ± SD of three biological replicates. Statistical significance was calculated using one-way ANOVA and Tukey multiple-comparisons.
