# Supplementary material for: Novel Syngeneic Cell Lines for Studying High-Risk BRAFV600E-Driven Colorectal Cancer In Vivo
Source: Cancer Res Commun. 2026 Feb 16;6(2):320–39. doi: 10.1158/2767-9764.CRC-25-0599 (PMC13037773; doi:10.1158/2767-9764.CRC-25-0599)
Supplement: Supplementary Figure S15 — shows membranes from the cytokine array and cell line–specific cytokine heatmaps of NaJa cells. [file crc-25-0599_supplementary_figure_s15_suppsf15.pdf]

## Supplementary Figure S15

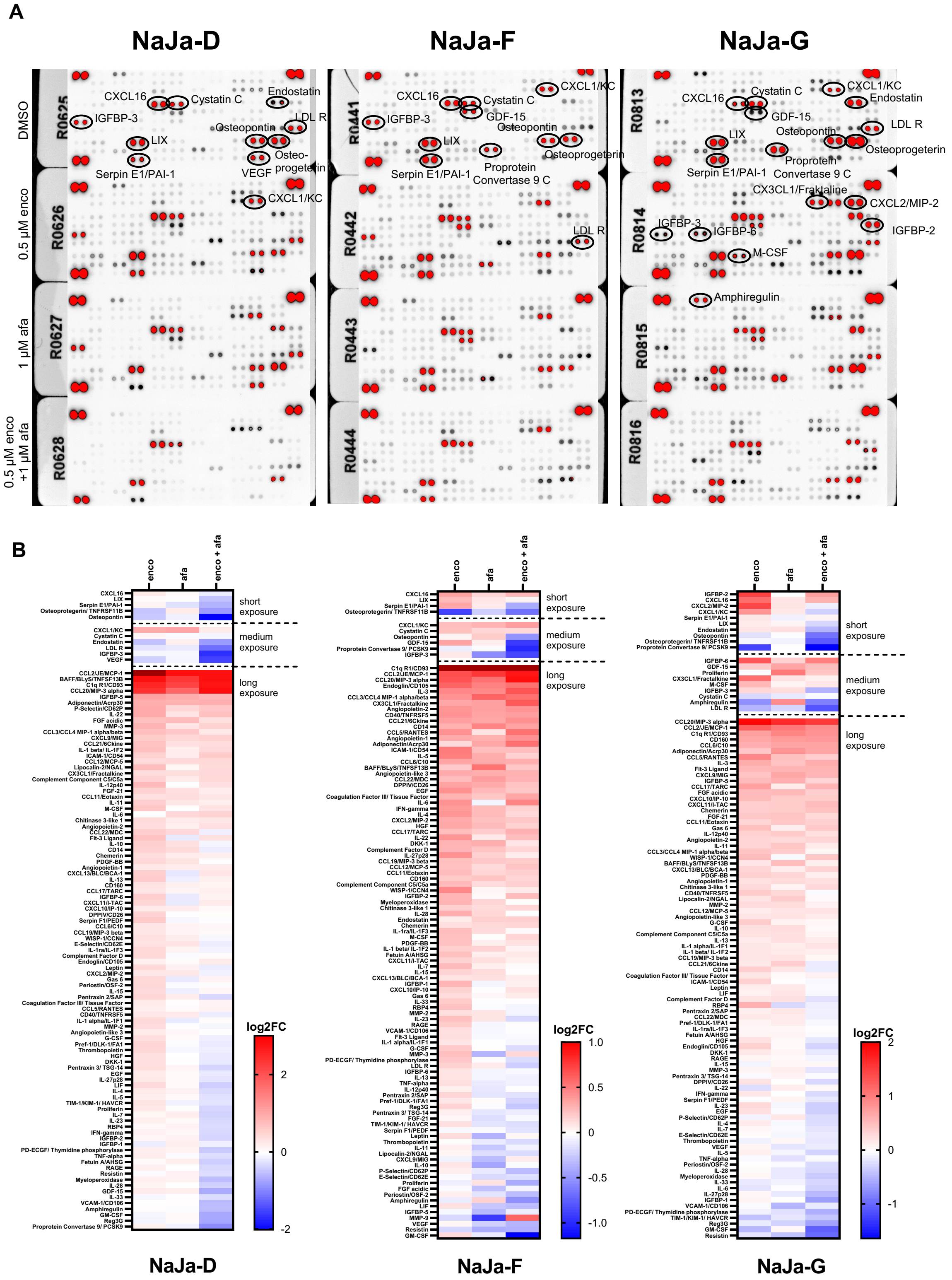


**Supplementary Figure S15. Detailed cytokine-array of NaJa cells.** (**A**) Cytokine array of NaJa cells treated either with DMSO or 0.5 µM encorafenib, 1 µM afatinib or both for five days. Shown are the membranes at the longest exposure time. The reference spots indicate that

the membrane was incubated with HRP-coupled phospho-tyrosine antibodies. (**B**) Heatmap of Cytokine arrays, clustered singly for each cell line.
