# Supplementary material for: Novel Syngeneic Cell Lines for Studying High-Risk BRAFV600E-Driven Colorectal Cancer In Vivo
Source: Cancer Res Commun. 2026 Feb 16;6(2):320–39. doi: 10.1158/2767-9764.CRC-25-0599 (PMC13037773; doi:10.1158/2767-9764.CRC-25-0599)
Supplement: Supplementary Figure S16 — shows a focus assay, Western blot and kinase assay characterizing the MC38-associated BRAF W450C mutation. [file crc-25-0599_supplementary_figure_s16_suppsf16.pdf]

Supplementary Figure S16

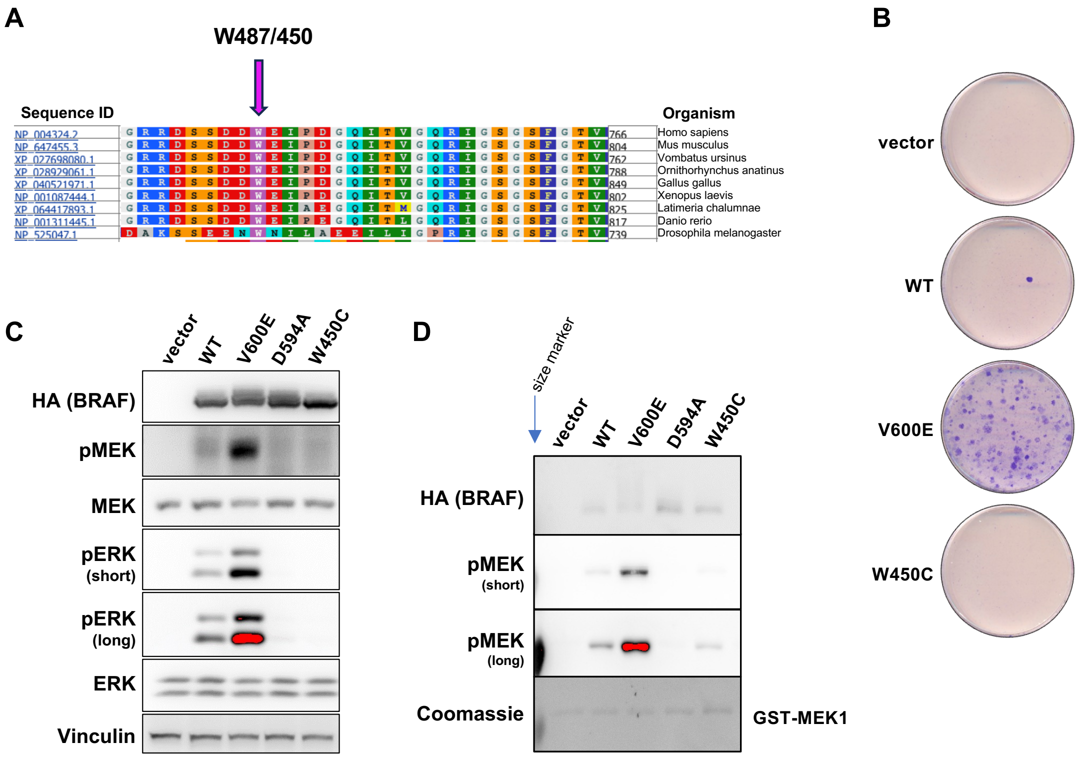

Supplementary Figure S16. The MC38 associated BRAF W450C mutation neither generates a hyperactive oncoprotein nor confers transforming potential. (**A**) Alignment showing the high conservation of W487, the residue mutated in MC38 cells, across BRAF orthologues form various vertebrate species and in *Drosophila* RAF. The indicated sequences were aligned using the COBALT tool (https://www.ncbi.nlm.nih.gov/tools/cobalt/re_cobalt.cgi). (**B**) Introduction of the equivalent W450C mutation into human BRAF does not generate a transforming oncoprotein. SV40 large T antigen immortalized murine embryonic fibroblasts (MEFs) were infected with either empty pMIG vector (negative control; vector) or its derivative encoding hemagglutinin (HA)-tagged wildtype BRAF (WT), V600E or the MC38 associated variant of unknown significance, W450C. Cells were grown to confluency, cultured for two weeks and stained with Giemsa solution as described previously (36). Note the absence of transformed foci in MEFs infected with pMIG/BRAF^{W450C}. (**C**) Western blot of total cell lysates (TCLs) of HEK293T cells transfected with either empty pMIG vector (negative control; vector) or pMIG/BRAF expression vectors encoding for wildtype BRAF (WT), V600E, the canonical kinase-dead BRAF mutant D594A or W450C. Detection of vinculin and MEK serve as loading control for pERK, total ERK represents the loading control for the anti-HA and pMEK staining. (**D**) *In vitro* kinase assay. HA-tagged BRAF was purified from the lysates shown in (C) by anti-HA antibody, washed extensively, diluted and subject to an *in vitro* kinase (IVK) reaction using

recombinant glutathionine-S-transferase (GST)-tagged MEK1 and ATP. IVK reactions were subject to Western blotting using pMEK antibodies. Addition of HA-tagged BRAF and GST-MEK1 substrate was confirmed by Western blotting and Coomassie staining of the PVDF membrane, respectively. Long exposures, as indicated by the red saturation signals in the lane with samples from $BRAF^{V600E}$ expressing cells were used to show lower levels of ERK and GST-MEK1 phosphorylation in (C) and (D), respectively.
